# Efficacy and safety of anisodine hydrobromide injection in acute ischemic stroke—a multicenter real-world observational study

**DOI:** 10.3389/fphar.2026.1806290

**Published:** 2026-05-21

**Authors:** Tianxiang Lan, Yajun Cheng, Tang Yang, Yuying Yan, Jingyu Cui, Shuai Jiang, Bo Wu

**Affiliations:** 1 Department of Neurology, West China Hospital, Sichuan University, Chengdu, China; 2 Center of Cerebrovascular Diseases, West China Hospital, Sichuan University, Chengdu, China

**Keywords:** acute ischemic stroke, anisodine hydrobromide, efficacy, observational study, treatment

## Abstract

**Objective:**

To evaluate the efficacy and safety of anisodine hydrobromide injection in patients with acute ischemic stroke in a real-world clinical setting.

**Design:**

Multicenter, real-world observational cohort study conducted from April 2018 to December 2021 with 90-day follow-up. Multivariable logistic regression and inverse probability of treatment weighting (IPTW) were applied.

**Participants:**

A total of 4,179 patients aged ≥18 years with acute ischemic stroke admitted within 30 days of onset were included; 2,120 received anisodine hydrobromide and 2,059 received best medical treatment.

**Exposure:**

Intravenous anisodine hydrobromide injection administered as part of routine clinical care.

**Outcomes:**

The primary outcome was unfavorable functional outcome (modified Rankin Scale [mRS] 3–6) at 90 days. Secondary outcomes included overall mRS distribution, changes in National Institutes of Health Stroke Scale (NIHSS) scores, hemorrhagic transformation, and 90-day mortality or recurrent stroke.

**Results:**

Unfavorable functional outcome occurred in 14.3% (282/2,120) of the anisodine group versus 20.6% (396/2059) of controls (adjusted OR, 0.63; 95% CI, 0.53–0.74). IPTW analysis confirmed the result (OR, 0.64; 95% CI, 0.51–0.80). The anisodine group had a more favorable mRS distribution (OR, 0.68; 95% CI, 0.61–0.77) and greater NIHSS improvement (mean ΔNIHSS difference = −0.61; 95% CI, −0.79 to −0.43). Hemorrhagic transformation, mortality, and recurrent stroke did not differ significantly between groups.

**Conclusion:**

In this large multicenter cohort conducted in China, anisodine hydrobromide showed a potential association with improved 90-day functional outcomes, with no clear increase in adverse events among patients with acute ischemic stroke.

**Clinical Trial Registration:**

https://www.chictr.org.cn/, identifier ChiCTR1900027517.

## Introduction

Stroke is the second leading cause of global disability-adjusted life-years (DALYs) and death, the trend expected to persist over the next 30 years (Vollset et al., 2024). In China, the prevalence rate of stroke is more than 1,000 cases per 100,000 people, with incidence rates continuing to rise annually. Acute ischemic stroke (AIS), the most common type of stroke, often results in severe sequalae and imposes great burden for patients and healthcare system (Wu et al., 2019; Saini et al., 2021; Feigin et al., 2024). Currently, the treatment options for AIS primarily include reperfusion and non-reperfusion therapies. Reperfusion therapy, encompassing intravenous thrombolysis and mechanical thrombectomy, represents the gold standard for treatment. However, its application is limited by a strict treatment time window, leading to low adoption rates. Data from 2019 to 2020 indicate that only 5.64% of AIS patients in China received intravenous thrombolysis, while a mere 1.45% underwent endovascular treatment (Ye et al., 2022). Consequently, conventional non-reperfusion therapies such as anti-platelet, anti-coagulation, neuroprotection, vasodilators, and statins remain the mainstay of clinical practice.

Anisodine hydrobromide, a compound extracted from the wild plant Hyoscyamine Tangut, interacts with muscarinic acetylcholine receptors (M1-M5). First investigated in the 20th century, it has demonstrated efficacy in treating retinal vascular disorders (Zhang et al., 2015). Preclinical and clinical studies have found that anisodine hydrobromide can penetrate the blood-brain barrier (BBB) and improve microcirculation, promoting the recovery of blood perfusion in ischemic brain tissue. It also has neuroprotective effects such as anti-inflammatory, antioxidative stress, attenuation of apoptosis, and inhibition of excitatory amino acids (Wang Y. et al., 2023; Jiang et al., 2023; Chen et al., 2017; Zeng et al., 2021). In 2023, China’s National Medical Products Administration approved anisodine hydrobromide for AIS-related acute paralysis, and it has since been widely adopted in clinical practice, with hundreds of thousands of treated patients. Although preliminary studies report promising outcomes (Zou et al., 2018; Wang et al., 2020; Dong Z. et al., 2021) large-scale clinical evidence regarding its efficacy and safety in AIS patients remains scarce. To address this gap, we collaborated with over 40 tertiary hospitals across China to conduct a multicenter, real-world observational study evaluating the therapeutic potential of anisodine hydrobromide in patients with AIS.

## Methods

### Study design

This is a prospective, multi-center, real-world, observational cohort study, following STROBE guideline, approved by the Ethics Committee on Biomedical Research, West China Hospital of Sichuan University (ethical approval document: No. 28 of 2018) and registered in the Chinese Clinical Trial Registry (ChiCTR1900027517). We conducted the research with 45 tertiary hospitals from more than 20 provinces in China. We enrolled patients admitted to the neurology wards of these hospitals. Standardized protocols were implemented for data collection, ensuring consistency and reliability across sites.

### Participants

We included patients aged 18 years and older with AIS and admitted to the hospitals within 30 days from the onset of symptoms between April 2018 and April 2021. All participants met the diagnostic criteria of Chinese guidelines for diagnosis and treatment of acute ischemic stroke 2018 (Neurology Branch of Chinese Medical Association CDG, 2018). Participants with acute intracranial hemorrhage, tumors, encephalitis, or other non-vascular intracranial lesions confirmed by neuroimaging (CT/MRI) were excluded. Additional exclusion criteria included pregnancy or breastfeeding, inability or unwillingness to complete follow-up, severe organ dysfunction, or life expectancy less than 3 months.

Participants could withdraw from the study under the following circumstances: voluntary withdrawal by the participant or a decision by the physician to discontinue the participant’s involvement for safety reasons. Data from these participants were excluded from the final analysis.

Prior to enrollment, all participants or their legal representatives were fully informed about the study’s purpose, procedures, potential risks, and possible benefits. Written informed consent was obtained voluntarily from all participants.

### Exposure

The researchers did not actively intervene in the decision to administer anisodine hydrobromide to participants. Instead, to better reflect real-world clinical practice, treatment decisions were guided by physicians’ clinical judgment based on an overall assessment of each participant’s condition rather than any single baseline characteristic. Participants who were administered to anisodine hydrobromide were categorized into the anisodine hydrobromide group, while those who did not receive the medication were placed in the best medical treatment group. Anisodine hydrobromide and other drugs used by the anisodine group and the best medical treatment group during hospitalization, and the dosage, route, course of drug use was all recorded.

### Data collection

Data were collected at five stages. Visit 1 (within 24 h of admission) included eligibility confirmation, consent, demographics, comorbidities, history, physical exam, neurological assessments (Glasgow score (GCS), National Institutes Health of Stroke Scale (NIHSS) score, modified Rankin Scale (mRS)), electrocardiogram (ECG), laboratory tests. Visits 2–3 (days 3 and 7) recorded vital signs, neurological scores, and adverse events. Visit 4 (discharge) included NIHSS, GCS, mRS, trial of. ORG 10172 in acute stroke treatment (TOAST) classification, treatments, and in-hospital events. Visit 5 (90 ± 14 days) assessed survival, mRS, recurrence, readmission, post-discharge therapy, and adverse events.

### Outcome

The primary outcomes are unfavorable functional outcome (mRS 3–6) at the 90-day from stroke onset. Secondary outcomes include the following: 1) distribution of mRS grade at 90-day; 2) change of NIHSS score from baseline to discharge or 30 days after symptom onset (ΔNIHSS = NIHSS score at discharge–NIHSS score at admission); 3) incidence of hemorrhagic transformation after ischemic stroke during hospitalization; 4) mortality during 90-day follow-up; 5) recurrence of stroke during 90-day follow-up.

Adverse events were collected using a combined passive and active surveillance approach. As a muscarinic cholinergic receptor antagonist, anisodine hydrobromide is associated with common side effects such as dry mouth, dizziness, blurred vision, urinary incontinence, fatigue (Lee et al., 2013; Liu et al., 2020). Thus, subjective symptoms reported by patients or caregivers, including dizziness, dry mouth, and fatigue, were recorded passively during routine clinical visits. In contrast, clinically significant adverse events of particular concern, such as tachycardia and severe delirium, were actively monitored and assessed by investigators during hospitalization. Major neurological and cardiovascular safety events were evaluated based on clinical examination and medical records.

### Statistical analysis

Analyses were restricted to participants who received treatment and had complete follow-up data. For continuous variables, we summarized the mean, standard deviation (SD), minimum, maximum, median, upper quartile, and lower quartile. For categorical variables, frequency and percentage were reported. Baseline data were analyzed using the Wilcoxon rank test or Cochran-Mantel-Haenszel (CMH) Chi-squared test, depending on the data type.

To evaluate the mRS score at 90 days, we first conducted a binary logistic regression analysis by defining mRS scores of 3–6 as unfavorable outcomes. We selected the following variables as covariates for adjustment: age, sex, smoking status, alcohol consumption, baseline NIHSS score, systolic blood pressure, TOAST classification, reperfusion therapy, use of antihypertensive agents, anticoagulants, lipid-lowering agents, antiplatelet agents, and hypoglycemic drugs, as well as laboratory values including creatinine, fasting glucose, serum lipid level, and onset-to-treatment time. We further performed ordinal logistic regression using the full distribution of mRS scores (0–6) to provide a more comprehensive evaluation of treatment efficacy. We applied inverse probability of treatment weighting (IPTW) (Austin and Stuart, 2015; Chesnaye et al., 2022; Yaghi et al., 2024) based on propensity scores to avoid bias caused by potential confounders: age, sex, smoking history, history of drinking alcohol, baseline NIHSS score, systolic blood pressure, TOAST classification, reperfusion treatment (intravenous thrombolysis, or endovascular treatment), concomitant medication, serum creatinine and glucose level, serum lipid level and time from symptom onset to admission. Covariate balance was assessed using standardized mean differences (SMDs) before and after inverse probability of treatment weighting. An absolute SMD < 0.1 was considered adequate balance. After obtaining the weighted data, we repeated both the binary and ordinal logistic regression analyses. Above statistical results were reported as odds ratio (OR) and 95% confidence interval (CI).

The mean differences (MD) and 95% CI of ΔNIHSS at each group were described, then using the paired t-test to compare ΔNIHSS between anisodine hydrobromide group and best medical treatment group. The incidence of hemorrhagic transformation during hospitalization, 90-day mortality, and stroke recurrence were statistically analyzed using the χ^2^ test or Fisher’s exact test.

Vital signs and ECG results recorded during treatment were analyzed as qualitative data, focusing on changes from baseline to each follow-up time point.

All primary analyses were conducted in the per-protocol (PP) population.

For the primary outcome, an additional intention-to-treat (ITT) analysis was performed as a supplementary analysis to assess the robustness of the results. In the ITT framework, the missing follow-up outcome data was primarily handled using multiple imputation under the missing-at-random assumption. Furthermore, a worst-case imputation strategy was applied as a sensitivity analysis within the ITT framework, in which the 90-day mRS is imputed as score 6. IPTW was also used in ITT and sensitivity analysis.

For the primary outcome, we additionally performed subgroup analyses stratified by age, sex, presence of hypertension, diabetes, hyperlipidemia, reperfusion therapy, baseline NIHSS score, and TOAST classification.

All hypothesis tests were two-sided, and test statistics with their corresponding *P* values were reported. Statistical significance was defined as *P* < 0.05. All statistical analyses were performed using SAS software (Version 9.4, SAS Institute Inc., Cary, NC, United States), and R software (Version 4.4.2).

## Results

### Baseline characteristics

We recruited participants between April 2018 and December 2021 from 45 hospitals in China. The anisodine hydrobromide group consisted of 2,120 participants, while the best medical treatment group included 2,059 participants. There were 286 censored cases (154 in the anisodine hydrobromide group and 132 in the best medical treatment group), resulting in a dropout rate of 6.8%. Most dropout occurred during follow-up. There were no significant differences in dropout rates between the two groups. Patients lost to follow-up showed comparable baseline disease severity and early neurological function to those who complete follow-up. Ultimately, 3,893 participants who had complete follow-up data were included in the final analysis, with 1,966 in the anisodine hydrobromide group and 1,927 in the control group. The mean age of the anisodine hydrobromide group was 63.9 ± 11.2 years, significantly younger than that of best medical treatment group at 64.8 ± 11.4 years. Anisodine hydrobromide group included 69.0% (n = 1,357) male, while 67.4% (n = 1,298) male enrolled in the best medical treatment group. A total of 116 participants (5.90%) in the anisodine hydrobromide group and 53 participants (2.75%) in the best medical treatment group received reperfusion therapy (*P* < 0.001). There was no significant difference found between two groups in baseline NIHSS score and mRS score, TOAST classification, comorbidities (hypertension, diabetes mellitus, hyperlipidemia, atrial fibrillation), history of transient ischemic attack (TIA). In the anisodine hydrobromide group, 94.6% of participants received low-dose therapy (mean 17.3 ± 5.5 mg/day for 8.7 ± 2.8 days). The middle-dose (22.0 ± 7.7 mg) and high-dose (26.5 ± 3.8 mg) groups were pooled with low-dose due to small sample sizes. Baseline characteristics of the two groups are shown in Table 1.

**TABLE 1:** Characteristics of participants at baseline.

Characteristics	Anisodine hydrobromide group (n = 1966)	Best medical treatment group (n = 1927)	P value
Age, mean (SD), y	63.88 (11.19)	64.83 (11.41)	<0.05
Sex, No. (%)	​	​	0.2648
Male	1,357 (69.02)	1,298 (67.36)	​
Female	609 (30.98)	629 (32.64)	​
Height, mean (SD), cm	167.78 (7.45)	167.65 (7.42)	0.6047
Weight, mean (SD), kg	68.08 (9.85)	68.05 (9.85)	0.7480
Race, No. (%)	​	​	<0.05
Han	1893 (96.29)	1878 (97.46)	​
Other	73 (3.71)	49 (2.54)	​
Smoke, No. (%)	853 (43.39)	810 (42.03)	0.3935
Drink, No (%)	634 (32.25)	545 (28.28)	<0.05
Systolic blood pressure, mean (SD), mmHg	147.82 (21.46)	149.50 (22.02)	0.0759
Diastolic blood pressure, mean (SD), mmHg	86.51 (13.34)	87.24 (13.84)	0.1257
Temperature, mean (SD), °C	36.47 (0.26)	36.46 (0.26)	0.3137
Heart rate, mean (SD), bpm	76.38 (10.27)	76.67 (9.91)	0.2964
Respiratory rate, mean (SD), bpm	18.67 (1.46)	18.68 (1.47)	0.9310
Hypertension, No. (%)	962 (48.93)	899 (46.65)	0.9404
Hyperlipidemia, No. (%)	79 (6.15)	74 (6.16)	0.9969
Diabetes mellitus, No. (%)	501 (39.02)	461 (38.35)	0.7334
TIA history, No. (%)	298 (23.21)	314 (26.12)	0.0919
Atrial fibrillation	34 (2.65)	37 (3.08)	0.5199
From onset to admission, mean (SD), hour	6.17 (0.09)	6.18 (0.09)	0.1140
NIHSS score, mean (SD)	4.97 (4.43)	4.77 (3.93)	0.4676
mRS score, mean (SD)	2.15 (1.32)	2.11 (1.31)	0.4067
GCS score, mean (SD)	​	​	​
E (Eye response)	3.91 (0.36)	3.94 (0.30)	<0.05
V (Verbal response)	4.78 (0.71)	4.79 (0.71)	0.4418
M (Motor response)	5.81 (0.67)	5.83 (0.66)	<0.05
Total	14.48 (1.52)	14.55 (1.40)	0.1427
TOAST, No. (%)	​	​	0.3726
Large artery atherosclerosis	1,057 (53.76)	1,069 (55.47)	​
Cardiogenic embolism	70 (3.56)	69 (3.58)	​
Small artery occlusion	646 (32.86)	635 (32.95)	​
Other clear causes	11 (0.56)	10 (0.52)	​
Unknown causes	182 (9.26)	144 (7.47)	​
TOAST, No. (%)	​	​	0.2475
Complete anterior circulation infarction	239 (12.16)	224 (11.62)	​
Partial anterior circulation infarction	737 (37.49)	693 (35.96)	​
Posterior circulation infarction	468 (23.80)	442 (22.94)	​
Lacunar infarction	522 (26.55)	568 (29.48)	​

Abbreviations: TIA, transient ischemic attack; NIHSS, national institutes of health stroke scale; mRS, modified rankin scale; GCS, glasgow coma scale; TOAST, trial of. ORG 10172 in acute stroke treatment, OAST, optimal acute stroke therapy.

Before propensity weighting, several baseline variables showed mild imbalance between groups. After applying IPTW using generalized boosted models with an average treatment effect in the overlap population (ATO) estimand, all covariates achieved excellent balance, with absolute SMDs below 0.1.

### Primary outcome

**FIGURE 1:**
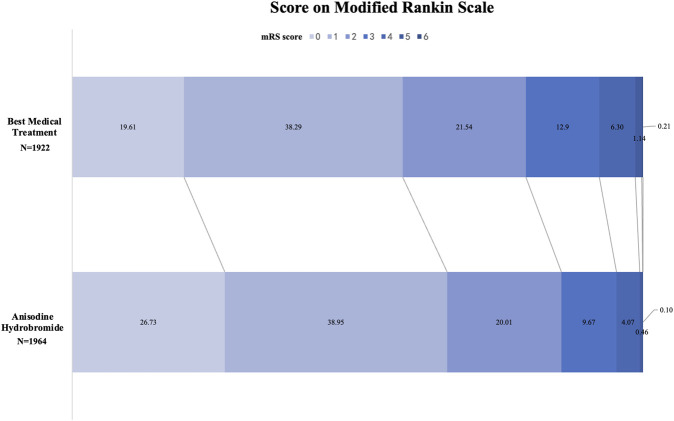
Distribution of mRS score at 90 days between best medical treatment group and anisodine hydrobromide group. The figure demonstrates the distribution of mRS score at 90 days between best medical treatment group and anisodine hydrobromide group. The proportion of mRS 0–2 in anisodine hydrobromide group was significantly higher than that in the best medical treatment group (85.69% and 79.44%).

The incidence of poor functional outcome was lower in the anisodine hydrobromide group (14.3%, n = 282) compared to the best medical treatment group (20.6%, n = 396). Binary logistic regression analysis suggested that, compared to the best medical treatment group, the anisodine hydrobromide had a 37% lower risk of poor functional outcomes (OR = 0.63, 95% CI: 0.53–0.74) (Figure 1). After applying IPTW, we conducted further analyses using binary logistic regression model based on the 90-day mRS outcomes. The weighted binary logistic regression revealed that anisodine hydrobromide treatment was associated with a lower likelihood of unfavorable functional outcomes compared to the best medical treatment group (OR = 0.64, 95% CI: 0.51–0.80).

### Secondary outcome

As shown in Table 1, Ordinal logistic regression demonstrated a more comprehensive analysis about the efficacy of anisodine, of which the anisodine group was associated with a shift toward better outcomes on the mRS scale at 90 days (OR = 0.68, 95% CI: 0.61–0.77). Similarly, after applying IPTW, the ordinal logistic regression showed a shift toward better mRS scores in the anisodine hydrobromide group (OR = 0.69, 95% CI: 0.58–0.81). The two groups had no significant difference in NIHSS score at baseline (5.0 ± 4.4 vs. 4.8 ± 4.0, *P* = 0.468). NIHSS score in anisodine hydrobromide group (2.9 ± 3.0) was lower than that of the best medical treatment group (3.3 ± 3.3) at discharge or 30 days from symptom onset (*P* < 0.001). The anisodine hydrobromide group exhibited a slightly greater reduction in NIHSS scores compared with the best medical treatment group (MD in ΔNIHSS = −0.61, 95% CI: -0.43 to −0.79).

Regarding hemorrhagic transformation after AIS during hospitalization, no significant difference was observed in anisodine hydrobromide group and best medical treatment group (0.3% vs. 0.4%, *P* = 0.567).

Moreover, no significant difference was observed between the anisodine hydrobromide and the best medical treatment groups in mortality (0.1% vs. 0.2%, *P* = 0.400) or recurrence of stroke (0.4% vs. 0.6%, *P* = 0.231) at 90 days.

**FIGURE 2:**
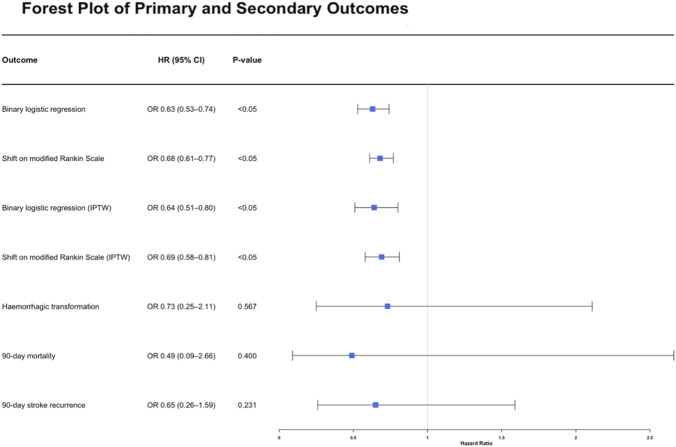
Forest plot demonstrates the odd ratio of primary and secondary outcomes. (CI: Confidential Interval; OR: Odds Ratio; mRS: modified Rankin Scale; IPTW: Inverse Probability of Treatment Weighting; NIHSS: National Institutes of Health Stroke Scale). This figure demonstrates that, whether considering the binary outcome or the overall mRS score distribution, patients in anisodine hydrobromide group achieved favored functional outcomes than those in best medical treatment group. Moreover, there were no significant differences between the two groups in terms of hemorrhagic transformation, long-term mortality, or stroke recurrence.

All primary and secondary results are shown in Table 2 and Figure 2.

**TABLE 2:** Primary and secondary outcomes between anisodine hydrobromide group and best medical treatment group.

Outcome	Anisodine hydrobromide	Best medical treatment	Effect (OR/MD)	*P* value
Poor functional outcome	14.3% (282/1972)	20.6% (396/1922)	6.2% (3.6%–8.6%)	<0.05
Binary logistic regression	​	​	0.63 (0.53–0.74)	<0.05
Distribution of mRS	​	​	0.68 (0.61–0.77)	<0.05
Binary logistic regression (IPTW)	​	​	0.64 (0.51–0.80)	<0.05
Distribution of mRS (IPTW)	​	​	0.69 (0.58–0.81)	<0.05
Baseline NIHSS score, mean (SD)	5.0 (4.4)	4.8 (4.0)	​	0.468
NIHSS at discharge/30days, mean (SD)	2.9 (3.0)	3.3 (3.3)	​	<0.05
Reduction in NIHSS (ΔNIHSS)	​	​	−0.61 (−0.43 to −0.79)	<0.05
Haemorrhagic transformation	0.3%	0.4%	0.73 (0.25–2.11)	0.567
90-day mortality	0.1%	0.2%	0.49 (0.09–2.66)	0.400
90-day stroke recurrence	0.4%	0.6%	0.65 (0.26–1.59)	0.231

mRS, modified Rankin Scale; IPTW, inverse probability of treatment weighting; MD, mean difference; NIHSS, national institutes of health stroke scale; OR, odds ratio.

### Safety outcomes

Anisodine hydrobromide was administered alongside other conventional treatments during hospitalization. The average onset time for side reaction is approximately 15–20 min after administration (Neuro-ophthalmology Group, 2020), and therefore, we monitored and recorded the adverse events during hospitalization. Among participants in the anisodine hydrobromide group, 9 cases of side effects were reported: 3 (0.15%) with dry mouth, 4 (0.19%) with dizziness, 1 (0.05%) with urinary incontinence, and 1 (0.05%) with fatigue. No side effects were reported in the best medical treatment group.

At each visit, we compared changes in vital signs, such as heart rate and blood pressure with the last visit within and between group. Most of the observed variations were not different, and those with significant difference were considered to have little clinical relevance. The ECG data of most participants (N = 2,965, 1,549 in anisodine group, 1,416 in best medical treatment group) was recorded as well. In the anisodine group, 63.2% of participants had normal ECG, with 11.6% abnormal but without clinical significance, and 25.3% abnormal with clinical significance. In best medical treatment group, the rate of normal, abnormal without clinical significance and abnormal with clinical significance was 67.0%, 10.2%, and 22.9%, respectively. However, no difference was found between the two groups in terms of ECG test (*P* = 0.093).

### ITT and sensitivity analysis

In the supplementary ITT analysis for the primary outcome, similar results were observed. Using multiple imputation, treatment with anisodine hydrobromide was associated with a lower likelihood of good functional outcome (adjusted OR, 0.71; 95% CI, 0.60–0.83). Consistent findings were obtained in the sensitivity analysis using the worst-case imputation strategy (adjusted OR, 0.72; 95% CI, 0.63–0.84), supporting the robustness of the results.

### Subgroup analysis

**FIGURE 3:**
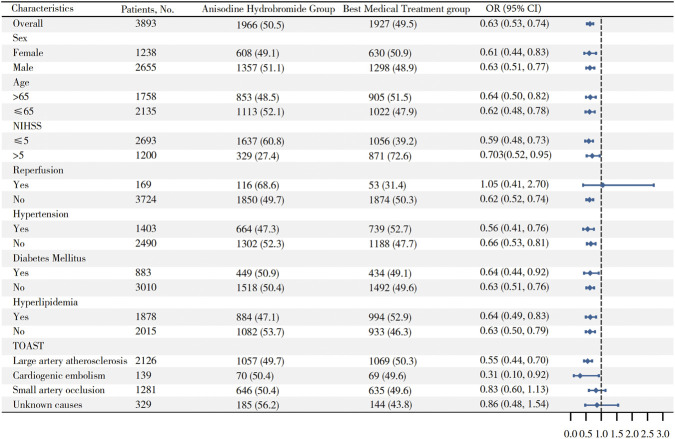
Subgroup analysis between anisodine hydrobromide and best medical treatment group. This figure showed that in most subgroup, anisodine hydrobromide group had a better outcome compared with best medical treatment group, except reperfusion, small artery occlusion and unknown cause subgroup. (NIHSS, National Institutes of Health Stroke Scale, TOAST, Trial of. ORG 10172 in Acute Stroke Treatment, OR, Odds Ratio).

Exploratory subgroup analyses yielded findings broadly consistent with those of the overall cohort, with anisodine hydrobromide generally associated with a lower risk of unfavorable outcomes across most prespecified subgroups, including age, sex, baseline NIHSS score, hypertension, diabetes, and hyperlipidemia. The treatment effect appeared to remain evident in the large artery atherosclerosis and cardiogenic embolism subgroup (Figure 3).

## Discussion

Our study provides large-scale, real-world evidence that anisodine hydrobromide may be associated with improved functional outcomes in patients with AIS compared with best medical treatment alone, while maintaining an acceptable safety profile. In this cohort, patients treated with anisodine hydrobromide demonstrated a higher proportion of favorable mRS score at 90 days. Additionally, treatment with anisodine hydrobromide was associated with a low incidence of reported adverse events.

The result is consistent with previous study on anisodine hydrobromide that also used mRS as key indicator for stroke recovery (Yu, 2022). Prior meta-analyses (Wang Y. et al., 2023), although including randomized controlled trials, were based on relatively small pooled sample sizes (approximately 1,300 patients), which may limit generalizability. By contrast, our multicenter, real-world analysis included a larger and more heterogeneous population, allowing assessment of treatment-associated outcomes across diverse clinical contexts, while still acknowledging the inherent limitations of observational data.

Although observational in nature, we applied IPTW based on propensity score to balance baseline characteristics between the anisodine group and the best medical treatment group, and the results remained consistent. However, unmeasured or residual confounding remains possible, including factors such as clinicians’ judgment in treatment selection, subtle differences in stroke severity not fully captured by baseline scales, variations in treatment timing, and center-level practice patterns. Moreover, due to the lack of detailed imaging data in the current dataset, we were unable to account for important factors such as infarct volume, collateral circulation, and other imaging markers of frailty. In addition, the observed imbalance in reperfusion therapy rates between groups may reflect underlying inter-center variability in resource availability and treatment practices, which could have influenced treatment allocation and clinical outcomes. These unobserved influences may have affected both treatment exposure and outcomes and cannot be fully corrected by statistical adjustment. The subgroup analyses were consistent with the primary results; however, no statistically significant effects were observed in certain subgroups with smaller sample sizes.

A notable characteristic of our cohort was the prolonged interval between symptom onset and hospital admission, resulting in a very low proportion of patients receiving intravenous thrombolysis or mechanical thrombectomy. This treatment pattern reflects the historical and regional context of stroke care during the study period and substantially influences the clinical framework in which functional outcomes should be interpreted. In contemporary stroke systems, where reperfusion therapy represents the standard of care, baseline prognosis, infarct progression, and determinants of recovery differ markedly from those observed in non-reperfusion populations (Elangwe et al., 2025). Therefore, the generalizability of our findings to settings with high reperfusion rates may be limited, and extrapolation to modern stroke care environments should be approached with caution. In addition, the small number of patients undergoing reperfusion therapy precluded subgroup analyses based on treatment modality, and the potential additive or synergistic effects of anisodine hydrobromide when combined with reperfusion therapy warrant further investigation.

The use of anisodine hydrobromide started in 24 h since admission. Other studies focusing on neuroprotection agents reported varying treatment windows, from 24 or 48 h in Edaravone (Fu et al., 2024) and butylphthalide (Guo et al., 2023), to 20 days in Qizhitongluo capsule (Yu et al., 2021). These differences highlight the need for future research to systematically evaluate the efficacy of anisodine hydrobromide across different treatment timepoints.

Currently, non-reperfusion treatments for AIS in clinical practice mainly include anticoagulation, antiplatelet, statins, neuroprotective drugs, and drugs that improve microcirculation. Common drugs that improve microcirculation include butylbenzidine (Wang A. et al., 2023) and human urinary kallidinogenase (HUK) (Ni et al., 2021). The former promotes angiogenesis by inducing the production of vascular growth factors such as vascular endothelial growth factor (VEGF) and regulating the hedgehog pathway (Dai et al., 2023). The later may enhance expression of VEGF and apelin/APJ (apelin receptor) through extracellular signal-regulated kinase 1 (ERK1) and ERK2 activation, as demonstrated in rat model (Han et al., 2015). Unlike butylbenzidine and HUK, anisodine hydrobromide regulates the autonomic nervous system by competing with acetylcholine for M-cholinergic receptor binding, thereby preventing vasoconstriction and vasospasm. Its mechanism, similar to atropine, blocks the M-cholinergic pathway but with a more favorable side effect profile (Liu et al., 2015; Wang et al., 2018). Beyond improving microcirculation, anisodine hydrobromide also plays a role in anti-inflammation, anti-oxidative stress, and inhibition of apoptosis. While of the precise mechanisms remains unclear, potential pathways include Ca^2+^ influx or modulation of cytokine release, requiring further study to explore (Wang Y. et al., 2023). Notably, despite strong biological plausibility, the history of acute ischemic stroke research has repeatedly shown that neuroprotective agents with compelling mechanistic rationale and preclinical efficacy have often failed to demonstrate clinical benefit in large randomized controlled trials (Dávalos et al., 2012; Shuaib et al., 2007). This discrepancy underscores the complexity of stroke pathophysiology and suggests that mechanistic promise alone is insufficient to ensure therapeutic effectiveness in real-world patients.

Against this backdrop, the mechanistic profile of anisodine hydrobromide should be interpreted as supportive rather than definitive. Its unique mode of action may nonetheless complement existing neuroprotective or microcirculation-enhancing strategies. Accordingly, combining anisodine hydrobromide with other neuroprotective drugs (e.g., Edaravone (Chen et al., 2021), ginkgolide (Dong Y. et al., 2021)) or microcirculation enhancers may produce synergistic therapeutic effects, potentially enhancing functional recovery of AIS patients. However, such combination strategies and their potential impact on functional recovery require confirmation in rigorously designed prospective randomized trials.

The comparable rates of hemorrhagic transformation and mortality rate between the two groups may reflect several considerations. Most patients were admitted beyond the standard treatment window (Hacke et al., 2008; Goyal et al., 2016), resulting in reduced use of reperfusion therapy—typically associated with higher hemorrhagic risk (Spronk et al., 2021) —and consequently lowering overall incidence. Ongoing studies are exploring whether extending this window may still yield benefits (Albers et al., 2024), which could support combining anisodine hydrobromide with thrombolysis or endovascular treatment. Furthermore, asymptomatic hemorrhagic transformation may have been underestimated due to the lack of timely neuroimaging. Additionally, the median NIHSS score was 4, suggesting that most patients had relatively mild stroke and thus a lower risk of mortality. Future studies should investigate the safety and efficacy of anisodine hydrobromide in patients with more severe strokes to better assess its potential benefits in high-risk populations.

This study has several limitations. First, treatment assignment in this study relied on physicians’ discretion rather than randomization, representing a fundamental limitation. Although multivariable adjustment was performed, physician decision-making is intrinsically influenced by nuanced and often unrecorded clinical factors—such as detailed infarct characteristics, subtle neurological findings, and overall comorbidity burden—that are difficult to fully capture using baseline variables alone. Consequently, substantial residual confounding related to treatment selection is unavoidable, which may bias the observed associations and limit the internal validity of the findings. These limitations underscore the need for well-designed randomized controlled trials to more definitively establish causal relationships. Second, less than 50% of patients had hypertension, which is lower than expected in AIS population (Lin et al., 2021; Wajngarten and Silva, 2019). Moreover, the median NIHSS score at baseline in our cohort was 4-5, representing relatively mild stroke severity. These may limit the generalizability of the findings and could potentially overestimate the treatment effect, but also leading to low rate of unfavorable outcome in both Anisodine and control groups compared with other real-world studies (Zhu et al., 2026). Third, patients in the anisodine hydrobromide group were slightly younger than controls, which may have influenced outcomes as younger patients usually have better outcomes. Additionally, the mean between-group difference in ΔNIHSS of −0.61 points, while statistically significant, is modest and its clinical relevance may be limited, particularly in a population predominantly composed of patients with mild stroke. As our cohort was mainly comprised of patients in the acute phase of ischemic stroke, the drug’s efficacy in subacute and chronic stages remains unknown. With respect to safety, adverse events were not collected through a standardized, prospective monitoring protocol. Instead, some were passively recorded in routine clinical documentation, while others were actively assessed. This inconsistency in data collection methods may have led to underreporting, particularly for mild or transient side effects. Lastly, since all participating centers were in China, international validation is needed to assess generalizability across different populations.

## Conclusion

To our knowledge, this study represents one of the most comprehensive real-world evaluations of anisodine hydrobromide in patients with AIS. The findings show reassuring safety and potential associations with favorable functional outcomes, especially in relatively mild stroke patients. Although the associations suggest potential neuroprotective effects, particularly in patients presenting beyond reperfusion windows, definitive conclusions regarding efficacy and optimal clinical use require confirmation in future randomized controlled trials.

## Data Availability

The original contributions presented in the study are included in the article/Supplementary Material, further inquiries can be directed to the corresponding authors.

## References

[B1] Albers G. W. Jumaa M. Purdon B. Zaidi S. F. Streib C. Shuaib A. (2024). Tenecteplase for stroke at 4.5 to 24 hours with perfusion-imaging selection. N. Engl. J. Med. 390 (8), 701–711. 10.1056/NEJMoa2310392 38329148

[B2] Austin P. C. Stuart E. A. (2015). Moving towards best practice when using inverse probability of treatment weighting (IPTW) using the propensity score to estimate causal treatment effects in observational studies. Stat. Med. 34 (28), 3661–3679. 10.1002/sim.6607 26238958 PMC4626409

[B3] Chen D. Peng C. Xie X. Chen Q. Liu H. Zhang S. (2017). Low dose of anisodine hydrobromide induced neuroprotective effects in chronic cerebral hypoperfusion rats. CNS Neurol. Disord. Drug Targets 16 (10), 1111–1119. 10.2174/1871527316666171026114043 29076436

[B4] Chen C. Li M. Lin L. Chen S. Chen Y. Hong L. (2021). Clinical effects and safety of edaravone in treatment of acute ischaemic stroke: a meta-analysis of randomized controlled trials. J. Clin. Pharm. Ther. 46 (4), 907–917. 10.1111/jcpt.13392 33638896 PMC8359409

[B5] Chesnaye N. C. Stel V. S. Tripepi G. Dekker F. W. Fu E. L. Zoccali C. (2022). An introduction to inverse probability of treatment weighting in observational research. Clin. Kidney J. 15 (1), 14–20. 10.1093/ckj/sfab158 35035932 PMC8757413

[B6] Dai M. Gui X. Jia S. Lv S. Dou H. Cui W. (2023). Dl-3-n-butylphthalide promotes angiogenesis in ischemic stroke mice through upregulating autocrine and paracrine sonic hedgehog. Acta Pharmacol. Sin. 44 (12), 2404–2417. 10.1038/s41401-023-01137-z 37580491 PMC10692133

[B7] Dávalos A. Alvarez-Sabín J. Castillo J. Díez-Tejedor E. Ferro J. Martínez-Vila E. (2012). Citicoline in the treatment of acute ischaemic stroke: an international, randomised, multicentre, placebo-controlled study (ICTUS trial). Lancet 380 (9839), 349–357. 10.1016/S0140-6736(12)60813-7 22691567

[B8] Dong Z. Han L. Duan P. Liang J. Dong R. Liu N. (2021). Anisodine hydrobromide injection for the treatment of acute ischemic stroke: a randomized controlled trial. J. Pharmacoepidemiol 30 (5), 296–300.

[B9] Dong Y. Zhang J. Wang Y. Zhao L. Li R. Wei C. (2021). Effect of Ginkgolide in Ischemic Stroke patients with large Artery Atherosclerosis: results from a randomized trial. CNS Neurosci. Ther. 27 (12), 1561–1569. 10.1111/cns.13742 34676982 PMC8611772

[B10] Elangwe K. C. Mathiesen E. B. Varmdal T. Indredavik B. Eltoft A. (2025). Trends in reperfusion treatments, functional outcomes and mortality for first-ever ischaemic stroke in Norway from 2014 to 2021: the Norwegian Stroke Registry. Eur. Stroke J. 10 (4), 1445–1453. 10.1177/23969873251331482 40221925 PMC11994638

[B11] Feigin V. L. Abate M. D. Abate Y. H. Abd ElHafeez S. Abd-Allah F. Abdelalim A. (2024). Global, regional, and national burden of stroke and its risk factors, 1990–2021: a systematic analysis for the Global Burden of Disease Study 2021. Lancet Neurology 23 (10), 973–1003. 10.1016/s1474-4422(24)00369-7 39304265 PMC12254192

[B12] Fu Y. Wang A. Tang R. Li S. Tian X. Xia X. (2024). Sublingual edaravone dexborneol for the treatment of acute ischemic stroke: the TASTE-SL randomized clinical trial. JAMA Neurol. 81 (4), 319–326. 10.1001/jamaneurol.2023.5716 38372981 PMC10877503

[B13] Goyal M. Menon B. K. van Zwam W. H. Dippel D. W. J. Mitchell P. J. Demchuk A. M. (2016). Endovascular thrombectomy after large-vessel ischaemic stroke: a meta-analysis of individual patient data from five randomised trials. Lancet 387 (10029), 1723–1731. 10.1016/S0140-6736(16)00163-X 26898852

[B14] Guo Z.-N. Yue B.-H. Fan L. Liu J. Zhu Y. Zhao Y. (2023). Effectiveness of butylphthalide on cerebral autoregulation in ischemic stroke patients with large artery atherosclerosis (EBCAS study): a randomized, controlled, multicenter trial. J. Cereb. Blood Flow & Metabolism 43 (10), 1702–1712. 10.1177/0271678X231168507 37021629 PMC10581234

[B15] Hacke W. Kaste M. Bluhmki E. Brozman M. Dávalos A. Guidetti D. (2008). Thrombolysis with alteplase 3 to 4.5 hours after acute ischemic stroke. N. Engl. Journal Medicine 359 (13), 1317–1329. 10.1056/NEJMoa0804656 18815396

[B16] Han L. Li J. Chen Y. Zhang M. Qian L. Chen Y. (2015). Human urinary kallidinogenase promotes angiogenesis and cerebral perfusion in experimental stroke. Plos One 10 (7), e0134543. 10.1371/journal.pone.0134543 26222055 PMC4519127

[B17] Jiang W. Shen J. Du X. Qiu Y. A. N. Zhong J. Ouyang Z. H. I. (2023). Anisodine hydrobromide alleviates oxidative stress caused by hypoxia/reoxygenation in human cerebral microvascular endothelial cells predominantly via inhibition of muscarinic acetylcholine receptor 4. Biocell 47 (10), 2255–2263. 10.32604/biocell.2023.030880

[B18] Lee K.-H. Morris-Natschke S. L. Brattlie J. Xie J. Belding E. (2013). Modern drug discovery from Chinese materia medica used in traditional Chinese medicine.

[B19] Lin X. Wang H. Rong X. Huang R. Peng Y. (2021). Exploring stroke risk and prevention in China: insights from an outlier. Aging (Albany NY) 13 (11), 15659–15673. 10.18632/aging.203096 34086602 PMC8221301

[B20] Liu W. D. Chen L. L. Shen C. Y. Jiang L. B. (2015). Neuroprotective effect of compound anisodine in a mouse model with chronic ocular hypertension. Chin. Med. J. Engl. 128 (19), 2652–2657. 10.4103/0366-6999.166043 26415805 PMC4736849

[B21] Liu Y. Wang L. Wan F. Yang N. (2020). Effects of anisodine hydrobromide on the cardiovascular and respiratory functions in conscious dogs. Drug Des. Devel Ther. 14, 4263–4276. 10.2147/DDDT.S268113 33116414 PMC7569038

[B22] Neuro-ophthalmology Group (2020). Chinese experts consensus on compound anisodine injection in the ischemic ophthalmopathy clinical practice (2020). Chin. J. Exp. Ophthalmol. 38, 553–561.

[B23] Neurology Branch of Chinese Medical Association CDG (2018). Neurology Branch, Chinese Medical Association. Chinese guidelines for diagnosis and treatment of acute ischemic stroke 2018. Chin. J. Neurology 51 (9), 666–682.

[B24] Ni J. Yao M. Wang L.-H. Yu M. Li R.-H. Zhao L.-H. (2021). Human urinary kallidinogenase in acute ischemic stroke: a single-arm, multicenter, phase IV study (RESK study). CNS Neurosci. & Ther. 27 (12), 1493–1503. 10.1111/cns.13724 34510762 PMC8611767

[B25] Saini V. Guada L. Yavagal D. R. (2021). Global epidemiology of stroke and access to acute ischemic stroke interventions. Neurology 97 (20), S6–s16. 10.1212/WNL.0000000000012781 34785599

[B26] Shuaib A. Lees K. R. Lyden P. Grotta J. Davalos A. Davis S. M. (2007). NXY-059 for the treatment of acute Ischemic stroke. N. Engl. J. Med. 357 (6), 562–571. 10.1056/NEJMoa070240 17687131

[B27] Spronk E. Sykes G. Falcione S. Munsterman D. Joy T. Kamtchum-Tatuene J. (2021). Hemorrhagic transformation in ischemic stroke and the role of inflammation. Front. Neurol. 12, 661955. 10.3389/fneur.2021.661955 34054705 PMC8160112

[B28] Vollset S. E. Ababneh H. S. Abate Y. H. Abbafati C. Abbasgholizadeh R. Abbasian M. (2024). Burden of disease scenarios for 204 countries and territories, 2022–2050: a forecasting analysis for the Global Burden of Disease Study 2021. Lancet 403 (10440), 2204–2256. 38762325 10.1016/S0140-6736(24)00685-8 PMC11121021

[B29] Wajngarten M. Silva G. S. (2019). Hypertension and stroke: update on treatment. Eur. Cardiol. 14 (2), 111–115. 10.15420/ecr.2019.11.1 31360232 PMC6659031

[B30] Wang S.-B. Yang X.-Y. Du G.-H. (2018). Anisodine natural small molecule drugs from plants. Singapore: Springer Singapore, 175–180.

[B31] Wang L. Mao L. Chen W. (2020). Clinical observation of anisodine hydrobromide injection in the treatment of acute cerebral infarction. Chin. Community Dr. 36 (31), 54–55.

[B32] Wang Y. Wan F. Hu P. He B. Hu Y. Liu Y. (2023). Efficacy and safety of anisodine hydrobromide injection for acute ischemic stroke: a systematic review and meta-analysis. Front. Pharmacol. 14, 1290755. 10.3389/fphar.2023.1290755 38034985 PMC10684921

[B33] Wang A. Jia B. Zhang X. Huo X. Chen J. Gui L. (2023). Efficacy and safety of butylphthalide in patients with acute ischemic stroke: a randomized clinical trial. JAMA Neurol. 80 (8), 851–859. 10.1001/jamaneurol.2023.1871 37358859 PMC10294018

[B34] Wu S. Wu B. Liu M. Chen Z. Wang W. Anderson C. S. (2019). Stroke in China: advances and challenges in epidemiology, prevention, and management. Lancet Neurology 18 (4), 394–405. 10.1016/S1474-4422(18)30500-3 30878104

[B35] Yaghi S. Shu L. Mandel D. Leon Guerrero C. R. Henninger N. Muppa J. (2024). Antithrombotic treatment for stroke prevention in cervical artery dissection: the STOP-CAD Study. Stroke 55 (4), 908–918. 10.1161/STROKEAHA.123.045731 38335240

[B36] Ye Q. Zhai F. Chao B. Cao L. Xu Y. Zhang P. (2022). Rates of intravenous thrombolysis and endovascular therapy for acute ischaemic stroke in China between 2019 and 2020. Lancet Regional Health – West. Pac. 21, 100406. 10.1016/j.lanwpc.2022.100406 PMC8873940 35243459

[B37] Yu Z. (2022). Effect and safety of anisodine hydrobromide injection for treating patients with acute ischemic stroke. Smart Healthc. 8 (08), 23–25.

[B38] Yu Y. Tang L. Cui F. Jiao F. Zhang D. Ma J. (2021). Effect of Qizhitongluo capsule on lower limb rehabilitation after stroke: a randomized clinical trial. Pharmacol. Res. 165, 105464. 10.1016/j.phrs.2021.105464 33515707

[B39] Zeng Y. Du X. Qiu Y. Jiang W. (2021). Anisodine hydrobromide alleviates hypoxia/reoxygenation (H/R)‐induced brain microvascular endothelial cell injury via muscarinic acetylcholine receptor 4. FASEB J. 35. 10.1096/fasebj.2021.35.s1.01722

[B40] Zhang M. Tian B. Wei W. (2015). Effect of compound anisodine on retinal function repair in diabetic retinopathy after panretinal photocoagulation. Zhonghua Shiyan Yanke Zazhi/Chinese J. Exp. Ophthalmol. 33, 155–158.

[B41] Zhu L. Qin Y. Kang T. Ying Y. Cao Y. Shi J. (2026). Discharge NIHSS scores are predictive of poor 3-month outcomes in patients with acute Ischemic stroke receiving intravenous thrombolysis. Ann. Med. 58 (1), 2610555. 10.1080/07853890.2025.2610555 41475420 PMC12777931

[B42] Zou Y. Zhang Y. Fei Y. Han X. Liu Y. (2018). The effect of hydrobromide anisodine on acute cerebral infarction. Stroke Nerv. Dis. 25 (4), 385–388.

