# Supplementary material for: Efficacy and safety of anisodine hydrobromide injection in acute ischemic stroke—a multicenter real-world observational study
Source: Front Pharmacol. 2026 May 21;17:1806290. doi: 10.3389/fphar.2026.1806290 (PMC13233479; doi:10.3389/fphar.2026.1806290)
Supplement: Supplementary file 1 [file Supplementaryfile1.docx]

**Efficacy and Safety of Anisodine Hydrobromide Injection in Acute Ischemic Stroke—A Multicenter Real-world Observational Study**

**Supplement materials**

| Version Number: | V1.0 |
| --- | --- |
| Version Date: | August 2025 |
| Investigator's Institution: | West China Hospital, Sichuan University |
| Department Responsible: | Department of Neurology |
| Contact Information: | TEL:18980602142  Email: dr.bowu@hotmail.com |
| Principal Investigators: | Bo Wu |
| Study Duration: | November 2019 — August 2025 |

| No. | Abbreviation | Full English name |
| --- | --- | --- |
| 1 | RCT | Randomised controlled trial |
| 2 | FDA | Food and Drug Administration |
| 3 | mRS | modified Rankin scale |
| 4 | MRI | Nuclear Magnetic Resonance Imaging |
| 5 | CT | Computer Tomography |
| 6 | CTA | Computer Tomographic Angiography |
| 7 | CTP | Computer Tomographic Perfusion |
| 8 | GCS | Glasgow Coma Scale |
| 9 | NIHSS | National Institute of Health Stroke Scale |
| 10 | TOAST | Trial of ORG 10172 in Acute Stroke Treatment |
| 11 | AE(s) | Adverse Event(s) |
| 12 | SC | Steering Committee |
| 13 | CCC | Central Coordinating Center |
| 14 | EDC | Electronic Data Capture |
| 15 | TIA | Transient Ischemic Attack |
| 16 | IPTW | Inverse Probability of Treatment Weighting |
| 17 | ECG | Electrocardiogram |
| 18 | MD | Mean Difference |
| 19 | OR | Odds Ratio |
| 20 | BBB | Blood-Brain Barrier |
| 21 | HDL | High-Density Lipoprotein |
| 22 | LDL | Low-Density Lipoprotein |
| 23 | TC | Total Cholesterol |
| 24 | TG | Triglycerides |

**Content**

[**PROTOCOL SYNOPSIS** 5](#_Toc227611346)

[**1.** **Background** 7](#_Toc227611347)

[**2.** **Study Objectives** 8](#_Toc227611348)

[**3.** **Study Design** 8](#_Toc227611349)

[**4.** **Patient Selection Procedure** 8](#_Toc227611350)

[4.1 Inclusion and Exclusion Criteria 8](#_Toc227611351)

[4.2 Withdrawal Criteria 9](#_Toc227611352)

[**5.** **Consent** 9](#_Toc227611353)

[**6.** **Intervention** 9](#_Toc227611354)

[**7.** **Data Collection** 10](#_Toc227611355)

[**8.** **Outcome** 11](#_Toc227611356)

[**9.** **Adverse Event** 11](#_Toc227611357)

[9.1 Definition of Adverse Events 11](#_Toc227611358)

[9.2 Adverse Event Monitoring and Reporting 12](#_Toc227611359)

[9.3 Adverse Event Management 12](#_Toc227611360)

[**10.** **Statistical Analysis** 12](#_Toc227611361)

[**11.** **Organization** 13](#_Toc227611362)

[1. Steering Committee (SC) 13](#_Toc227611363)

[2. Central Coordinating Centre (CCC) 13](#_Toc227611364)

[3. Research Design and Execution 14](#_Toc227611365)

[4. Data Monitoring and Management 14](#_Toc227611366)

[5. Participating Center 14](#_Toc227611367)

[**12.** **Ethics and Privacy** 17](#_Toc227611368)

[**13.** **Result** 17](#_Toc227611369)

[1.Baseline characteristics 17](#_Toc227611370)

[2. Drug usage 20](#_Toc227611371)

[3. Primary Outcome 20](#_Toc227611374)

[4. Secondary Outcomes 21](#_Toc227611375)

[5. Safety outcomes 22](#_Toc227611376)

[6. ITT and sensitivity analysis 29](#_Toc227611377)

[7. Subgroup analyses 29](#_Toc227611378)

[**14.** **Funding** 30](#_Toc227611379)

[**15.** **Reference** 30](#_Toc227611380)

### **PROTOCOL SYNOPSIS**

| Study Objective | 1. To analyze the progression, complications, and short- and long-term outcomes of patients with different levels of acute ischemic stroke in a real-world setting;  2. To analyze the effects of anisodine hydrobromide and other treatments (including antiplatelet, anticoagulant, and dehydration) on the short- and long-term outcomes of patients with acute ischemic stroke in a real-world setting. |
| --- | --- |
| Study Design | Prospective, multicenter registry study, cohort study. This study is a real-world observational study and does not interfere with daily clinical practice. |
| Sample Size | A total of 5000 patients with acute ischemic stroke were enrolled consecutively; 2500 of them were treated with anisodine hydrobromide (anisodine hydrobromide group) and 2500 were not treated with anisodine hydrobromide (best medical treatment group). |
| Participants | Inclusion Criteria:  1) Patients admitted with a diagnosis of ischemic stroke (meeting the diagnostic criteria for ischemic stroke in the ‘Guidelines for the Diagnosis and Treatment of Acute Ischemic Stroke in China 2018’ [1]) who underwent CT/MRI to exclude cerebral hemorrhage;  2) Age ≥ 18 years, regardless of gender;  3) Interval between onset and admission ≤ 30 days;  4) Patients or their family members provided informed consent for the study and signed the informed consent form. |
|  | Exclusion Criteria:  1) Patients with acute intracranial hemorrhage, tumors, encephalitis, or other non-vascular intracranial lesions confirmed by neuroimaging (CT/MRI);  2) Pregnant or lactating women;  3) Patients with poor compliance, inability to complete, or refusal to complete follow-up;  4) Patients with severe dysfunction of vital organs;  5) Patients with critical illness and a life expectancy of less than three months. |
| Group | Anisodine hydrobromide group and best medical treatment group. |
| Intervention | All patients received routine treatment based on their clinical history, symptom profile, and other specific circumstances, in accordance with the "Guidelines for the Diagnosis and Treatment of Acute Ischemic Stroke in China 2018". All medications, including the name, route of administration, dosage, and course of treatment, were recorded during hospitalization.  Anisodine hydrobromide Group:  Anisodine Hydrobromide Injection was divided into three dosage group:  Low Dose: Intravenous infusion; 2 mg once daily. Course of treatment: 7-14 days.  Median Dose: Intravenous infusion; 2 mg twice daily. Course of treatment: 7-14 days.  High Dose: Intravenous infusion; ≥ 3 mg once or twice daily. Course of treatment: 7-14 days. |
| Study Endpoints | Primary outcome:   1. unfavorable functional outcome (mRS 3–6) at the 90-day from stroke onset; 2. distribution of mRS grade at 90 days   Secondary outcomes:   1. change of NIHSS score from baseline to discharge or 30 days after symptom onset (ΔNIHSS = NIHSS score at discharge – NIHSS score at admission); 2. incidence of hemorrhagic transformation after ischemic stroke during hospitalization; 3. death during follow-up; 4. recurrence of stroke during follow-up.   Safety outcomes:   1. Occurrence of adverse cardiovascular events (heart rate, blood pressure) during medication; 2. Adverse reactions during medication: dry mouth, dizziness, flushing, blurred vision/mydriasis, urinary incontinence, fatigue, jaundice, confusion; 3. Vital signs during medication (temperature, pulse, respiration, blood pressure); 4. Electrocardiogram examination during medication; 5. 5) Laboratory tests during medication (blood routine, blood sugar, blood lipids, liver and kidney function, myocardial enzymes, blood electrolytes, coagulation function). |

### **Background**

Stroke refers to a syndrome of limited or global brain dysfunction caused by acute cerebral circulatory disturbances. The "China Stroke Prevention and Treatment Report 2021" indicates that the incidence, prevalence, mortality, and per capita hospitalization medical expenses of ischemic stroke in my country are on the rise. In 2019, the incidence was 145 per 100,000 people and the prevalence was 1,256 per 100,000 people [2]. The crude mortality rate for stroke was 158.63 per 100,000 people in rural areas and 129.41 per 100,000 people in urban areas. The ‘Guidelines for the Diagnosis and Treatment of Acute Ischemic Stroke in China 2023’ indicate that the three-month disability rate after acute ischemic stroke is 14.6%-23.1%, and the one-year disability rate is 13.9%-14.2% [3]. This high disability rate imposes a significant medical and economic burden on the country.

Acute ischemic stroke (also known as acute cerebral infarction) is the most common type of stroke, accounting for approximately 60-80% of all stroke cases. It is characterized by high morbidity, mortality, disability, and recurrence rates. However, effective treatments for acute ischemic stroke are very limited. Current guidelines still primarily recommend reperfusion therapy (thrombolysis and arterial embolectomy) as a Level I recommendation [3].

Reperfusion therapy faces various limitations in practical application, including its narrow time window for implementation; numerous contraindications and the risk of hemorrhagic transformation; low thrombolysis rates (5.6%) and thrombectomy rates (1.4%) for ischemic stroke in China [4]; and, even with complete recanalization of the occluded vessel, approximately 50% of patients still fail to recover functional independence due to microcirculatory dysfunction and other factors. This limits the number of patients who can benefit and requires additional medications for supplemental treatment. Furthermore, it is particularly noteworthy that patients with severe cerebral infarction, who have the worst prognosis and are the most difficult to treat, are often excluded from clinical research. The efficacy and safety of reperfusion therapy in this unique population has long been understudied. Non-reperfusion therapy is still the main clinical treatment method. Current drugs mostly focus on a single stage and a single target. However, drug development and clinical treatment for cerebral infarction should focus on multi-stage (pathogenesis, during and after reperfusion therapy, rehabilitation therapy, etc.) and multi-target brain cell protection.

Anisodine hydrobromide, a compound extracted from the wild plant Hyoscyamine tangut, interacts with muscarinic acetylcholine receptors (M1-M5). Research on anisodine started since last century, previous studies have applied it to retinal diseases and have shown good efficacy [5].

Existing basic and clinical studies have found that anisodine hydrobromide can penetrate the blood-brain barrier (BBB) and improve microcirculation, promoting the recovery of blood perfusion in ischemic brain tissue. It also has neuroprotective effects such as anti-inflammatory, antioxidative stress, attenuation of apoptosis, and inhibition of excitatory amino acids [6] [7] [8] [9]. Anisodine hydrobromide received approval for active pharmaceutical ingredient and formulation production in 1981. In 2002, the preparation was reissued with a national approval number, and in 2013 the API was granted a new approval number. In 2016, related formulations were again approved for marketing. On October 31, 2023, the National Medical Products Administration approved a revision of its indications: from ‘vascular headache, retinal vasospasm, ischemic optic neuropathy, acute paralysis, Parkinsonian tremor, and as an antidote for organophosphorus pesticide poisoning’ to ‘vascular headache, retinal vasospasm, ischemic optic neuropathy, and acute paralysis caused by acute ischemic stroke’.

To date, anisodine hydrobromide injection has been used in more than 350,000 patients in China, with no reports of fatal adverse events. This project aims to first conduct a real-world observational clinical study to provide the foundation for subsequent high-quality randomized controlled trials (RCTs). Real-world observational research objectively records patients’ diagnostic and therapeutic regimens and clinical outcomes without interfering with clinical decision-making. Compared with RCTs, its advantages include closer alignment with routine clinical practice, broader inclusion criteria, and the ability to incorporate large sample sizes, thereby more accurately reflecting a drug’s effectiveness and safety in real-world medical settings. Consequently, real-world evidence has recently been recognized by the U.S. FDA, and the global adoption of real-world studies has become a new trend.

Given the well-established mechanisms of action of anisodine hydrobromide and its extensive clinical use in patients with acute ischemic stroke, applying real-world clinical research methods to further evaluate its efficacy and safety in this population has substantial reference value for rational clinical use and for the design of future RCTs. Therefore, this study adopts a prospective, multicenter registry and cohort design based on real-world settings, enrolling patients with acute ischemic stroke to investigate the effectiveness and safety of anisodine hydrobromide injection in routine clinical practice.

### **Study Objectives**

1. To analyze the progression, complications, and short- and long-term outcomes of patients with different levels of acute ischemic stroke in a real-world setting;

2. To analyze the effects of anisodine hydrobromide and other treatments (including antiplatelet, anticoagulant, and dehydration) on the short- and long-term outcomes of patients with acute ischemic stroke in a real-world setting.

### **Study Design**

Prospective, multicenter registry study, cohort study. This study is a real-world observational study and does not interfere with daily clinical practice.

### **Patient Selection Procedure**

4.1 Inclusion and Exclusion Criteria

Inclusion and exclusion criteria were demonstrated in Table 1.

Table 1. Inclusion and exclusion criteria

| Inclusion Criteria:  1) Patients admitted with a diagnosis of ischemic stroke (meeting the diagnostic criteria for ischemic stroke in the "Guidelines for the Diagnosis and Treatment of Acute Ischemic Stroke in China 2018") who underwent CT/MRI to exclude cerebral hemorrhage;  2) Age ≥ 18 years, regardless of gender;  3) Interval between onset and admission ≤ 30 days;  4) Patients or their family members provided informed consent for the study and signed the informed consent form. | Exclusion Criteria:  1) Patients with acute intracranial hemorrhage, tumors, encephalitis, or other non-vascular intracranial lesions confirmed by neuroimaging (CT/MRI);  2) Pregnant or lactating women;  3) Patients with poor compliance, inability to complete, or refusal to complete follow-up;  4) Patients with severe dysfunction of vital organs;  5) Patients with critical illness and a life expectancy of less than three months. |
| --- | --- |

4.2 Withdrawal Criteria

1. The patient withdraws informed consent;
2. The principal investigator (or attending physician), for safety reasons or based on the patient’s specific clinical condition, determines that the patient should discontinue participation in the study.

### **Consent**

Researchers will inform the patient about the research and seek their or their legal guardian's signed informed consent before the study. The communication process will be recorded. To minimize instances of consent withdrawal, patients for whom a paper copy of the informed consent cannot be obtained prior to the start of the intervention should be excluded.

5.1 Patient Consent

The patient signs the informed consent form directly, and the researcher provides the patient with the relevant information.

5.2 Legal Guardian Consent

If the patient is unable to sign the informed consent form personally, the patient's legal guardian shall sign on their behalf. This signatory should be the same as the signatory of other surgical documents.

5.3 Withdrawal of Consent

The information statement provided to the patient and/or their next of kin or proxy should clearly state that the patient may withdraw from the study at any time without any obligation or explanation. Such withdrawal should be recorded in the patient's file.

### **Intervention**

The best medical treatment group received conventional treatment, including but not limited to antiplatelet therapy, lipid-lowering therapy, blood pressure control, blood sugar control, oxygen inhalation, rehabilitation therapy and other drugs and physical comprehensive therapy.

In the drug group, excepting conventional treatment, anisodine hydrobromide infusion was administered within 24 hours of patient admission. According to the dosing regimen, patients were divided into high-dose, medium-dose, and low-dose subgroups. 1）Low dose: Intravenous infusion, 2 mg once daily. Duration: 7–14 consecutive days. 2）Medium dose: Intravenous infusion, 2 mg twice daily. Duration: 7–14 consecutive days. 3）High dose: Intravenous infusion, ≥3 mg once or twice daily. Duration: 7–14 consecutive days.

### **Data Collection**

Study Visits and Assessments

Visit 1 (within 24 hours of admission; Day 0)

1. Obtain written informed consent.
2. Verify inclusion and exclusion criteria.
3. Record demographic data (age, sex, ethnicity, occupation, smoking history, alcohol consumption).
4. Document medical history (stroke, hypertension, diabetes mellitus, dyslipidemia, coronary artery disease, atrial fibrillation, etc.).
5. Record medication history within the past month.
6. Document stroke onset and admission details (time of onset, main symptoms; NIHSS score; GCS score; mRS score).
7. Vital signs: body temperature, pulse, respiration, blood pressure.
8. Electrocardiogram (ECG).
9. Laboratory tests: complete blood count, blood glucose, lipid profile, liver and renal function, cardiac biomarkers*, serum electrolytes, coagulation function (*optional).
10. Imaging: head CT and/or MRI + CTA* and/or CTP* (*optional).

Hospitalization Period

Visit 2 (Day 3 after admission)

1. Vital signs (body temperature, pulse, respiration, blood pressure).
2. NIHSS score; GCS score.
3. Repeat laboratory tests* (complete blood count, blood glucose, lipid profile, liver and renal function, cardiac biomarkers, serum electrolytes, coagulation function) (*optional).
4. Repeat imaging* (head CT and/or MRI) (*optional).
5. Record adverse events (AEs).

Visit 3 (Day 7 after admission)

1. Vital signs (body temperature, pulse, respiration, blood pressure).
2. NIHSS score; GCS score.
3. Repeat laboratory tests* (same as Visit 2) (*optional).
4. Repeat imaging* (head CT and/or MRI) (*optional).
5. Record adverse events.

Visit 4 (at discharge or Day 30 after admission)

1. Vital signs (body temperature, pulse, respiration, blood pressure).
2. NIHSS score; GCS score; mRS score.
3. Repeat laboratory tests in at least 500 patients (complete blood count, blood glucose, lipid profile, liver and renal function, cardiac biomarkers, serum electrolytes, coagulation function).
4. Repeat imaging examinations (at least 150 patients with ≥2 MRI scans; at least 150 patients with repeat CTA and/or CTP).
5. TOAST classification.
6. Record in-hospital treatment regimens (pharmacological treatment, standard Western rehabilitation, traditional Chinese medicine–based rehabilitation, surgical intervention).
7. Record adverse events.

Follow-up Period

Visit 5 (Day 90 ± 14 after stroke onset)

1. Survival status (for deceased patients, record date and cause of death).
2. mRS score.
3. Stroke recurrence (document occurrence and time).
4. Readmission (number and reasons).
5. Post-discharge treatment (pharmacological therapy, standard Western rehabilitation, traditional Chinese medicine–based rehabilitation, surgical intervention).
6. Record adverse events.

### **Outcome**

Primary outcome:

1. Unfavorable functional outcome (mRS 3–6) at the 90-day from stroke onset;
2. Distribution of mRS grade at the 90-day from stroke onset.

Secondary outcomes:

1. change of NIHSS score from baseline to discharge or 30 days after symptom onset (ΔNIHSS = NIHSS score at discharge – NIHSS score at admission);
2. incidence of hemorrhagic transformation after ischemic stroke during hospitalization;
3. death during follow-up;
4. recurrence of stroke during follow-up.

Safety outcomes:

1. Occurrence of adverse cardiovascular events (heart rate, blood pressure) during medication;
2. Adverse reactions during medication: dry mouth, dizziness, flushing, blurred vision/mydriasis, urinary incontinence, fatigue, jaundice, confusion;
3. Vital signs during medication (temperature, pulse, respiration, blood pressure);
4. Electrocardiogram examination during medication;
5. Laboratory tests during medication (blood routine, blood sugar, blood lipids, liver and kidney function, myocardial enzymes, blood electrolytes, coagulation function).

### **Adverse Event**

9.1 Definition of Adverse Events

An adverse event (AE) is defined as any untoward medical occurrence in a patient or clinical investigation subject administered a pharmaceutical product, which does not necessarily have a causal relationship with the treatment. AEs include increased fatigue, fits or seizures, worsening vision or visual difficulties, increasing frequency or severity of headaches, accidents (e.g. falls) or injuries [10]. In our study, cardiovascular adverse events were also monitored by tracking heart rate and blood pressure. These events included arrhythmias (such as sinus tachycardia, sinus bradycardia, atrial flutter, atrial fibrillation, atrioventricular block, paroxysmal supraventricular tachycardia, ventricular tachycardia, and ventricular fibrillation), as well as abnormal blood pressure fluctuations beyond the normal range, defined as systolic blood pressure <90 mmHg or >140 mmHg, and diastolic blood pressure <60 mmHg or >90 mmHg. The ECG outcomes are divided into three group 1) normal: no abnormalities detected; 2) abnormal but not clinically significant: deviations from normal ECG patterns that were judged by the investigator as not affecting clinical management or patient safety; 3) abnormal and clinically significant: ECG abnormalities that were considered clinically relevant, requiring medical attention, influencing treatment decisions, or posing potential safety concerns.

9.2 Adverse Event Monitoring and Reporting

Monitoring and reporting of AEs should begin at the time of grouping and continue until the end of the follow-up period. All AEs must be followed up on until resolution within 90 days post-procedure or until the condition stabilizes, is resolved, or results in death. All AEs must be promptly recorded in the EDC system, including details such as the start and end dates of the event, severity, relationship to the study intervention, treatments administered, and outcomes.

9.3 Adverse Event Management

In the case of a SAE, immediate standardized treatment should also be provided, and the principal investigator at the center should determine whether the trial needs to be terminated.

### **Statistical Analysis**

Efficacy analysis was conducted for all participants who entered the cohort and completed follow-up. For continuous variables, we summarized the mean, standard deviation (SD), minimum, maximum, median, upper quartile, and lower quartile. For categorical variables, frequency and percentage were reported. Baseline data were analyzed using the Wilcoxon rank test or Cochran-Mantel-Haenszel (CMH) Chi-squared test, depending on the data type.

To evaluate the mRS score at 90 days, we first conducted a binary logistic regression analysis by defining mRS scores of 3–6 as unfavorable outcomes. We selected the following variables as covariates for adjustment: age, sex, smoking status, alcohol consumption, baseline NIHSS score, systolic blood pressure, TOAST classification, reperfusion therapy, use of antihypertensive agents, anticoagulants, lipid-lowering agents, antiplatelet agents, and hypoglycemic drugs, as well as laboratory values including creatinine, fasting glucose, high-density lipoprotein (HDL), low-density lipoprotein (LDL), total cholesterol (TC), triglycerides (TG), and onset-to-treatment time. We further performed ordinal logistic regression using the full distribution of mRS scores (0–6) to provide a more comprehensive evaluation of treatment efficacy. We applied inverse probability of treatment weighting (IPTW) [11] [12, 13] based on propensity scores to avoid bias caused by potential confounders: age, sex, smoking history, history of drinking alcohol, baseline NIHSS score, systolic blood pressure, TOAST classification, reperfusion treatment (intravenous thrombolysis, or endovascular treatment), concomitant medication (antihypertensive drugs, hypoglycemic drugs, lipid-lowering agents, anticoagulants, antiplatelet drugs), serum creatinine and glucose level, serum lipid level including (HDL, LDL-C, TC, TG), and time from symptom onset to admission. After obtaining the weighted data, we repeated both the binary and ordinal logistic regression analyses. Above statistical results were reported as odds ratio (OR) and 95% confidence interval (CI).

The mean differences (MD) and 95% CI of ΔNIHSS at each group were described, then using the paired t-test to compare ΔNIHSS between anisodine hydrobromide group and best medical treatment group. The incidence of hemorrhagic transformation during hospitalization, 90-day mortality, and stroke recurrence were statistically analyzed using the χ² test or Fisher's exact test.

Laboratory test results before and after treatment were evaluated based on clinical judgment categories: normal, abnormal without clinical significance and abnormal with clinical significance. Changes in abnormal and positive results before and after treatment were summarized using cross-tabulations. For cases with abnormalities at the end of treatment, a detailed description of results and clinical significance was provided in a tabulated format, distinguishing between clinically significant and non-significant findings.

Vital signs and ECG results recorded during treatment were analyzed as qualitative data, focusing on changes from baseline to each follow-up time point.

All primary analyses were conducted in the per-protocol (PP) population.

For the primary outcome, an additional intention-to-treat (ITT) analysis was performed as a supplementary analysis to assess the robustness of the results. In the ITT framework, the missing follow-up outcome data was primarily handled using multiple imputation under the missing-at-random assumption. Furthermore, a worst-case imputation strategy was applied as a sensitivity analysis within the ITT framework, in which the 90-day mRS is imputed as score 6. IPTW was also used in ITT and sensitivity analysis.

For the primary outcome, we additionally performed subgroup analyses stratified by age, sex, presence of hypertension, diabetes, hyperlipidemia, reperfusion therapy, baseline NIHSS score, and TOAST classification.

All hypothesis tests were two-sided, and test statistics with their corresponding *P* values were reported. Statistical significance was defined as *P* < 0.05. All statistical analyses were performed using SAS software (Version 9.4, SAS Institute Inc., Cary, NC, USA), and R software (Version 4.4.2).

### **Organization**

This multicenter study was organized under the leadership of **West China Hospital, Sichuan University**, which served as the principal coordinating institution.

1. Steering Committee (SC)

The Steering Committee, composed of senior neurologists and clinical trial experts from West China Hospital and selected participating institutions, was responsible for defining the overall study objectives and clinical relevance. It reviewed and approved the study protocol and any subsequent amendments, provided strategic oversight of trial conduct, and ensured strict adherence to ethical and regulatory standards. In addition, the Steering Committee guided the dissemination of study results and maintained the scientific integrity of the trial throughout its course.

2. Central Coordinating Centre (CCC)

The Central Coordinating Centre, West China Hospital, Sichuan University, undertook the overall operational management of the trial. It coordinated communication among all participating centers, organized investigator meetings and training programs, and monitored recruitment progress across sites. The CCC was also responsible for ensuring quality control in data collection and reporting and acted as the primary liaison with both the study sponsor and regulatory authorities, thereby ensuring consistency and compliance across the multicenter collaboration.

3. Research Design and Execution

The study protocol and statistical analysis plan were developed by the core research team at West China Hospital, with an emphasis on methodological rigor and standardization. This team was responsible for establishing the clinical trial design, defining eligibility criteria, and selecting appropriate outcome measures. They also implemented detailed procedures for patient enrollment, treatment allocation, and follow-up, while ensuring uniform application of the protocol across all sites. Standardized investigator training was conducted to guarantee protocol compliance, and consistent guidelines were applied for adverse event reporting and safety monitoring.

4. Data Monitoring and Management

An independent Data Monitoring Committee (DMC) (Beijing Bioknow Information Technology Co., Ltd.) provided oversight of patient safety and data integrity by regularly reviewing recruitment progress, adverse events, and adherence to the study protocol. When necessary, the DMC evaluated interim safety and efficacy outcomes and issued recommendations regarding the continuation, modification, or termination of the trial, ensuring that no undue risks were imposed on participants. In parallel, data management was carried out by an independent professional unit, which designed and maintained the electronic data capture (EDC) system, performed centralized data checks and consistency reviews, and managed queries with site investigators. The data management team also conducted data cleaning, coded adverse events, and finalized database lock before the statistical analysis, thereby guaranteeing the reliability and accuracy of the trial data.

5. Participating Center

More than 40 tertiary hospitals across China participated as collaborating centers. All participating centers were required to meet the following criteria:

1）A tertiary hospital or a neurology inpatient ward/stroke unit of equivalent qualification, with the capacity to continuously admit patients with AIS;

2）Approval from the institutional ethics committee and ability to obtain written informed consent from patients or their legal representatives;

3）Compliance with real-world study standards and AE reporting requirements;

4）Capability to complete four scheduled in-hospital visits plus a 90 ± 14 day follow-up (outpatient or telephone), with documentation of readmission, recurrence, modified Rankin Scale (mRS), and other clinical outcomes;

5）Adequate safety management for study medication: ability to identify contraindications of anisodine hydrobromide according to the prescribing information, administer intravenous infusion properly, record dosage/duration and concomitant medications, and perform AE/serious adverse event (SAE) monitoring and reporting;

6）A research team trained and certified in standardized administration and inter-rater calibration of NIHSS, mRS, and GCS assessments;

7）Availability of electronic data capture (EDC) systems/database entry and quality control capacity.

All centers that met the requirements, participated in and completed the study are listed in Table 2. The number of patients in the anisodine hydrobromide group and best medical treatment group at the completion of the study in each center is shown in Table 3

Table 2. Hospital number and name of each participating center

| **Center No.** | **Name of Each Participating Center** |
| --- | --- |
| 01 | **West China Hospital, Sichuan University** |
| 02 | **Zhongshan Hospital, Xiamen University** |
| 03 | **The Affiliated of Taizhou People's Hospital** of Nanjing Medical University |
| 04 | **Fuzhou Second General Hospital** |
| 05 | **The First People's Hospital of Zhaoqing** |
| 06 | **Shijiazhuang People's Hospital** |
| 07 | **Hebei Medical University Third Hospital** |
| 08 | **Daqing Oilfield General Hospital** |
| 11 | **The First Affiliated Hospital of Hebei North University** |
| 12 | Changzhou NO.2 People's Hospital |
| 13 | **Zhangjiakou First Hospital** |
| 14 | **Affiliated Hospital of Guangdong Medical University** |
| 15 | **The Third Affiliated Hospital of Sun Yat‑sen University** |
| 16 | **Chengdu Fifth People's Hospital** |
| 17 | Liaoning Province Health Industry Group **Bengang General Hospital** |
| 21 | **The First Affiliated Hospital of Nanchang University** |
| 22 | **Jiangxi Provincial People's Hospital** |
| 23 | Liaoning Province Health Industry Group **Fukuang General Hospital** |
| 24 | **The Second Affiliated Hospital of Nanchang University** |
| 26 | **The Second Affiliated Hospital of Chengdu Medical College Nuclear Industry Hospital No. 416 Hospital of China Nuclear Industry** |
| 27 | **Hospital of Chengdu University of Traditional Chinese Medicine** |
| 29 | **Chengdu First People's Hospital** |
| 30 | West China Fourth Hospital of Sichuan University |
| 31 | Sichuan Science City Hospital |
| 32 | **Chengdu Third People's Hospital** |
| 33 | **Panzhihua Central Hospital** |
| 34 | **Dandong Central Hospital** |
| 35 | **The First Affiliated Hospital of Henan University of Chinese Medicine** |
| 36 | **The First Affiliated Hospital of Henan University** |
| 37 | **Huaihe Hospital of Henan University** |
| 38 | **Kaifeng Central Hospital** |
| 40 | **Fuxin Central Hospital** |
| 41 | **The First Affiliated Hospital of China Medical University** |
| 42 | **Central Hospital Affiliated to Shenyang Medical College** |
| 43 | **Benxi Central Hospital** |
| 44 | Tianjin Medical University General Hospital |
| 46 | **Xinxiang First People's Hospital** |
| 48 | **Guangdong Sanjiu Brain Hospital** |
| 49 | **Jinjiang Hospital** |
| 51 | **Ansteel Group General Hospital** |
| 52 | **The First Affiliated Hospital of Jinzhou Medical University** |
| 53 | **Hebei Medical University Fourth Hospital** |
| 55 | Liaoning Province Health Industry Group Fuxin Mine General Hospital |
| 58 | **The Second People's Hospital of Gansu Province** |
| 63 | **Leshan People's Hospital** |

Table 3. The number of patients in the anisodine hydrobromide group and best medical treatment group in each center

| **Center No.** | **Anisodine Hydrobromide Group** | **Best Medical Treatment Group** | **Total** |
| --- | --- | --- | --- |
| 1 | 25 | 43 | 68 |
| 2 | 127 | 106 | 233 |
| 3 | 61 | 85 | 146 |
| 4 | 35 | 33 | 68 |
| 5 | 44 | 81 | 125 |
| 6 | 38 | 42 | 80 |
| 7 | 70 | 70 | 140 |
| 8 | 20 | 38 | 58 |
| 11 | 53 | 92 | 145 |
| 12 | 53 | 53 | 106 |
| 13 | 56 | 13 | 69 |
| 14 | 54 | 38 | 92 |
| 15 | 18 | 23 | 41 |
| 16 | 31 | 50 | 81 |
| 17 | 29 | 28 | 57 |
| 21 | 47 | 26 | 73 |
| 22 | 41 | 35 | 76 |
| 23 | 184 | 95 | 279 |
| 24 | 51 | 28 | 79 |
| 26 | 62 | 49 | 111 |
| 27 | 76 | 75 | 151 |
| 29 | 21 | 24 | 45 |
| 30 | 12 | 15 | 27 |
| 31 | 29 | 37 | 66 |
| 32 | 30 | 22 | 52 |
| 33 | 14 | 13 | 27 |
| 34 | 10 | 9 | 19 |
| 35 | 36 | 72 | 108 |
| 36 | 38 | 16 | 54 |
| 37 | 29 | 17 | 46 |
| 38 | 86 | 25 | 111 |
| 40 | 35 | 39 | 74 |
| 41 | 55 | 65 | 120 |
| 42 | 41 | 35 | 76 |
| 43 | 37 | 48 | 85 |
| 44 | 28 | 29 | 57 |
| 46 | 19 | 2 | 21 |
| 48 | 36 | 77 | 113 |
| 49 | 11 | 39 | 50 |
| 51 | 27 | 20 | 47 |
| 52 | 42 | 69 | 111 |
| 53 | 34 | 40 | 74 |
| 55 | 79 | 83 | 162 |
| 58 | 26 | 17 | 43 |
| 63 | 16 | 11 | 27 |
| Total | 1966 | 1927 | 3893 |

### **Ethics and Privacy**

This study was conducted in accordance with the ethical principles of the Declaration of Helsinki and followed all applicable national and local regulatory requirements. Prior to patient enrollment, each participating center was required to obtain written approval from its Institutional Ethics Committee or equivalent authority. The principal investigator at each site was responsible for reporting any protocol amendments, deviations, SAEs, or routine safety updates to the corresponding ethics committee in a timely manner.

Patient confidentiality and privacy were strictly safeguarded throughout the study. Source documents containing personally identifiable information were accessible only to authorized investigators, data collectors, and monitors for verification purposes. Data transmitted to the CCC were anonymized and identified solely by participant study numbers. Details of data confidentiality and privacy protection were explicitly described in the patient information sheet and reinforced through the informed consent process.

### **Result**

1.Baseline characteristics

We recruited participants between April 2018 and December 2021 from 45 tertiary hospitals. The anisodine hydrobromide group consisted of 2,120 participants, while the best medical treatment group included 2,059 participants. There were 286 censored cases (154 in the anisodine hydrobromide group and 132 in the best medical treatment group), resulting in a dropout rate of 6.8%. There was no significant difference in the dropout rate between the two groups. The primary reason for dropout was early discharge or loss to follow-up after discharge. Ultimately, 3,893 participants were included in the final analysis, with 1,966 in the anisodine hydrobromide group and 1,927 in the control group. The mean (±SD) age of the anisodine hydrobromide group was 63.9 (±11.2) years, significantly younger than that of best medical treatment group at 64.8 (±11.4) years (P<0.05). Anisodine hydrobromide group included 69.0% (n = 1357) male, while 67.4% (n=1298) male enrolled in the best medical treatment group. The proportion of Han participants in anisodine hydrobromide group (96.3%) was significantly lower compared with the best medical treatment group (97.5%) (*P* < 0.05). Moreover, the proportion of participants consuming alcohol in the anisodine hydrobromide group (32.3%) was higher than that of best medical treatment group (28.3%) (*P* < 0.05). At baseline, the eye response score of the GCS had significant differences between anisodine hydrobromide group and best medical treatment group, with 3.91 ± 0.36 and 3.94 ± 0.30, respectively. Among them, 116 (5.90%) and 53 (2.75%) participants received reperfusion therapy in anisodine hydrobromide group and best medical treatment group (*P* < 0.05) There was no significant difference found between two groups in baseline NIHSS score and mRS score, TOAST classification, comorbidities (hypertension, diabetes mellitus, hyperlipidemia, atrial fibrillation), history of transient ischemic attack (TIA). Baseline characteristics of the two groups are shown in Table 4.

Table 4. Characteristics of Participants at Baseline

|  | Anisodine hydrobromide group (n =1966) | Best medical treatment group  (n = 1927) | *P* value |
| --- | --- | --- | --- |
| Age, y | 63.88(11.19) | 64.83(11.41) | 0.0091 |
| Sex, No. (%) |  |  | 0.2648 |
| Male | 1357(69.02) | 1298(67.36) |  |
| Female | 609(30.98) | 629(32.64) |  |
| Height, cm | 167.78±7.45 | 167.65±7.42 | 0.6047 |
| Weight, kg | 68.08±9.85 | 68.05±9.85 | 0.7480 |
| Race, No. (%) |  |  | 0.0362 |
| Han | 1893(96.29) | 1878(97.46) |  |
| Other | 73(3.71) | 49(2.54) |  |
| Smoke, No. (%) | 853(43.39) | 810(42.03) | 0.3935 |
| Drink, No (%) | 634(32.25) | 545(28.28) | 0.0071 |
| Systolic blood pressure, mmHg | 147.82±21.46 | 149.50±22.02 | 0.0759 |
| Diastolic blood pressure, mmHg | 86.51±13.34 | 87.24±13.84 | 0.1257 |
| Temperature, ℃ | 36.47±0.26 | 36.46±0.26 | 0.3137 |
| Heart rate, bpm | 76.38±10.27 | 76.67±9.91 | 0.2964 |
| Respiratory rate, bpm | 18.67±1.46 | 18.68±1.47 | 0.9310 |
| Hypertension, No. (%) | 962(48.93) | 899(46.65) | 0.9404 |
| Hyperlipidemia, No. (%) | 79(6.15) | 74(6.16) | 0.9969 |
| Diabetes mellitus | 501(39.02) | 461(38.35) | 0.7334 |
| TIA history, No. (%) | 298(23.21) | 314(26.12) | 0.0919 |
| Atrial fibrillation | 34(2.65) | 37(3.08) | 0.5199 |
| From onset to admission, min | 22229±338.42 | 22244±333.15 | 0.1140 |
| NIHSS score | 4.97±4.43 | 4.77±3.93 | 0.4676 |
| mRS score | 2.15±1.32 | 2.11±1.31 | 0.4067 |
| GCS score |  |  |  |
| **E (Eye response)** | 3.91±0.36 | 3.94±0.30 | 0.0015 |
| **V (Verbal response)** | 4.78±0.71 | 4.79±0.71 | 0.4418 |
| **M (Motor response)** | 5.81±0.67 | 5.83±0.66 | 0.0222 |
| Total | 14.48±1.52 | 14.55±1.40 | 0.1427 |
| TOAST, No. (%) |  |  | 0.3726 |
| Large artery atherosclerosis | 1057(53.76) | 1069(55.47) |  |
| Cardiogenic embolism | 70(3.56) | 69(3.58) |  |
| Small artery occlusion | 646(32.86) | 635(32.95） |  |
| Other clear causes | 11(0.56) | 10(0.52) |  |
| Unknown causes | 182(9.26) | 144(7.47) |  |
| TOAST, No. (%) |  |  | 0.2475 |
| Complete anterior circulation infarction | 239(12.16) | 224(11.62) |  |
| Partial anterior circulation infarction | 737(37.49) | 693(35.96) |  |
| Posterior circulation infarction | 468(23.80) | 442(22.94) |  |
| Lacunar infarction | 522(26.55) | 568(29.48) |  |
| Abbreviations: TIA, transient ischemic attack, NIHSS, national institutes of health stroke scale, mRS, modified rankin scale, GCS, Glasgow coma scale, TOAST, trial of. ORG 10172 in acute stroke treatment, OAST, optimal acute stroke therapy. | | | |

## 2. Drug usage

## The treatment group was divided into three dosing regimens. Dose group 1 received 17.32 ± 5.52 mg for 8.66 ± 2.76 days; dose group 2 received 21.96 ± 7.71 mg; and dose group 3 received 26.46 ± 3.76 mg (details shown in Table 5).

## The sample size of dose group 1 was 1,859 cases (94.61%), dose group 2 had 93 cases (4.73%), and dose group 3 had 13 cases (0.66%). Since dose groups 2 and 3 together accounted for only 5.39% of the total population and their dosing amounts were higher than group 1, no separate statistical analyses were conducted for groups 2 and 3. Instead, groups 2 and 3 were combined with group 1 for pooled analysis, which did not have a significant impact on the statistical results.

Table 5. The dosage record in anisodine hydrobromide group

|  | Low Dosage | Median Dosage | High Dosage |
| --- | --- | --- | --- |
| N(Missing) | 1859(1) | 93(0) | 13(0) |
| Mean±SD | 17.32±5.52 | 21.96±7.71 | 26.46±3.76 |
| Median | 14.00 | 20.00 | 27.00 |
| Q1, Q3 | 14.00,20.00 | 16.00,28.00 | 24.00,28.00 |
| Min, Max | 4.00,74.00 | 12.00,46.00 | 21.00,33.00 |

3. Primary Outcome

At 90 days, the incidence of poor functional outcome (mRS 3–6) was significantly lower in the anisodine hydrobromide group compared with the best medical treatment group (14.3% [n=282] vs 20.6% [n=396]). This corresponded to an absolute risk reduction of 6.2% (MD = 6.2%, 95% CI: 3.6%–8.6%, P < 0.05).

Binary logistic regression analysis indicated that anisodine hydrobromide was associated with a 37% reduction in the risk of poor functional outcome compared with best medical treatment (OR = 0.63, 95% CI: 0.53–0.74, P < 0.05). Ordinal logistic regression further demonstrated a significant shift toward better functional outcomes on the mRS scale at 90 days (OR = 0.68, 95% CI: 0.61–0.77, P < 0.05).

After applying inverse probability of treatment weighting (IPTW) to account for potential confounders, the results remained consistent. Weighted binary logistic regression showed that anisodine hydrobromide significantly reduced the likelihood of unfavorable outcomes (OR = 0.64, 95% CI: 0.51–0.80, P < 0.05). Similarly, IPTW-adjusted ordinal logistic regression confirmed a favorable shift in mRS distribution (OR = 0.69, 95% CI: 0.58–0.81, P < 0.05).

4. Secondary Outcomes

Baseline NIHSS scores did not differ significantly between the two groups (5.0 ± 4.4 vs 4.8 ± 4.0, P = 0.468). At discharge or 30 days, patients treated with anisodine hydrobromide had lower NIHSS scores compared with the best medical treatment group (2.9 ± 3.0 vs 3.3 ± 3.3, P < 0.05). The reduction in NIHSS from baseline was greater in the anisodine hydrobromide group (mean difference ΔNIHSS = –0.61, 95% CI: –0.79 to –0.43, P < 0.05).

Regarding in-hospital complications, the incidence of hemorrhagic transformation did not differ between the anisodine hydrobromide and best medical treatment groups (0.3% vs 0.4%, P = 0.567). At 90 days, no significant between-group differences were observed in mortality (0.1% vs 0.2%, P = 0.400) or stroke recurrence (0.4% vs 0.6%, P = 0.231).


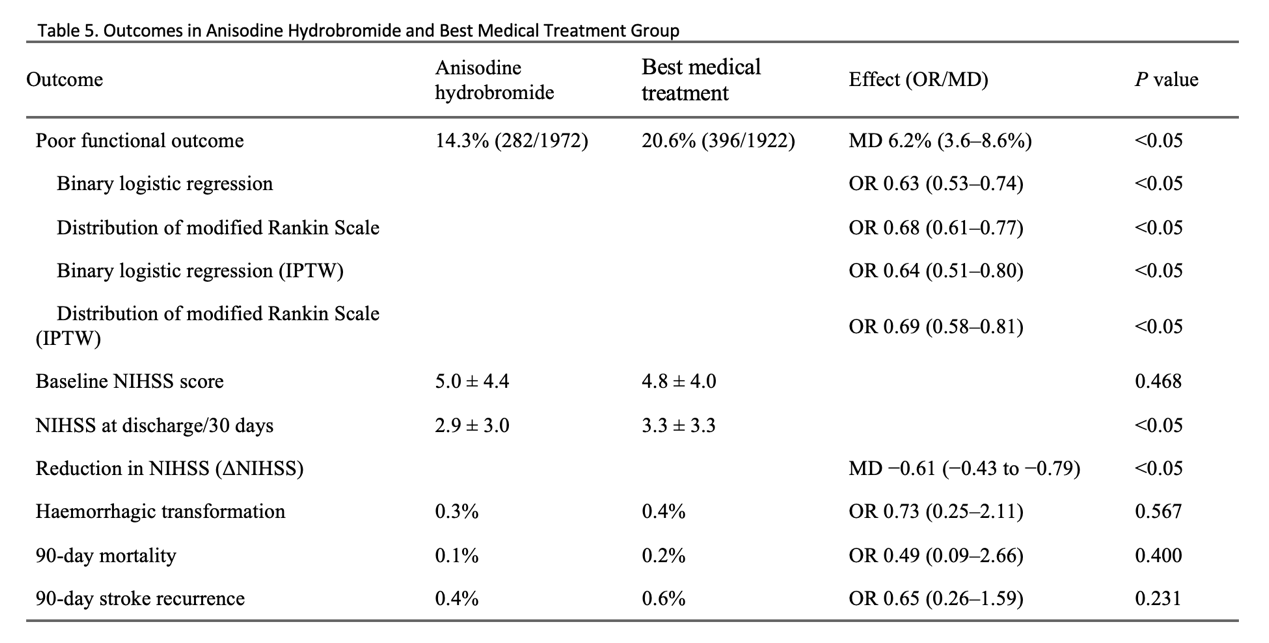


Figure 1. Distribution of mRS score at 90 days between best medical treatment group and anisodine hydrobromide group


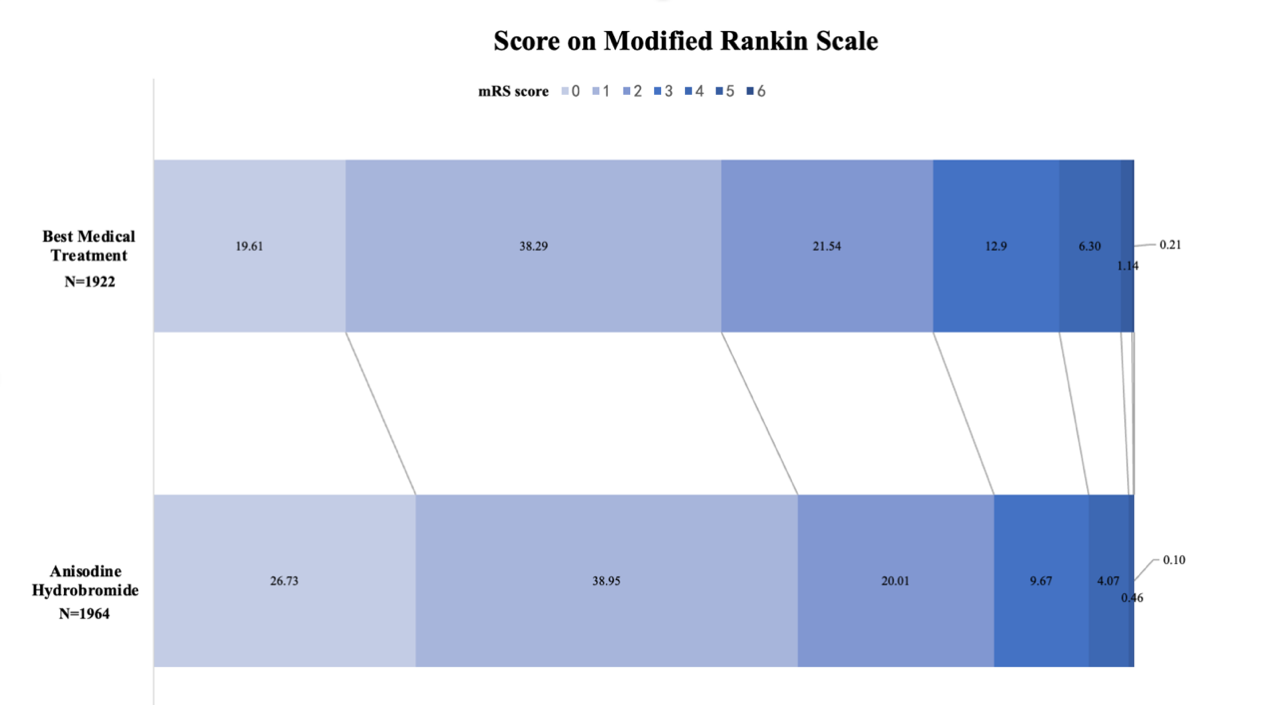


Figure 2. Forest plot of primary and secondary outcomes


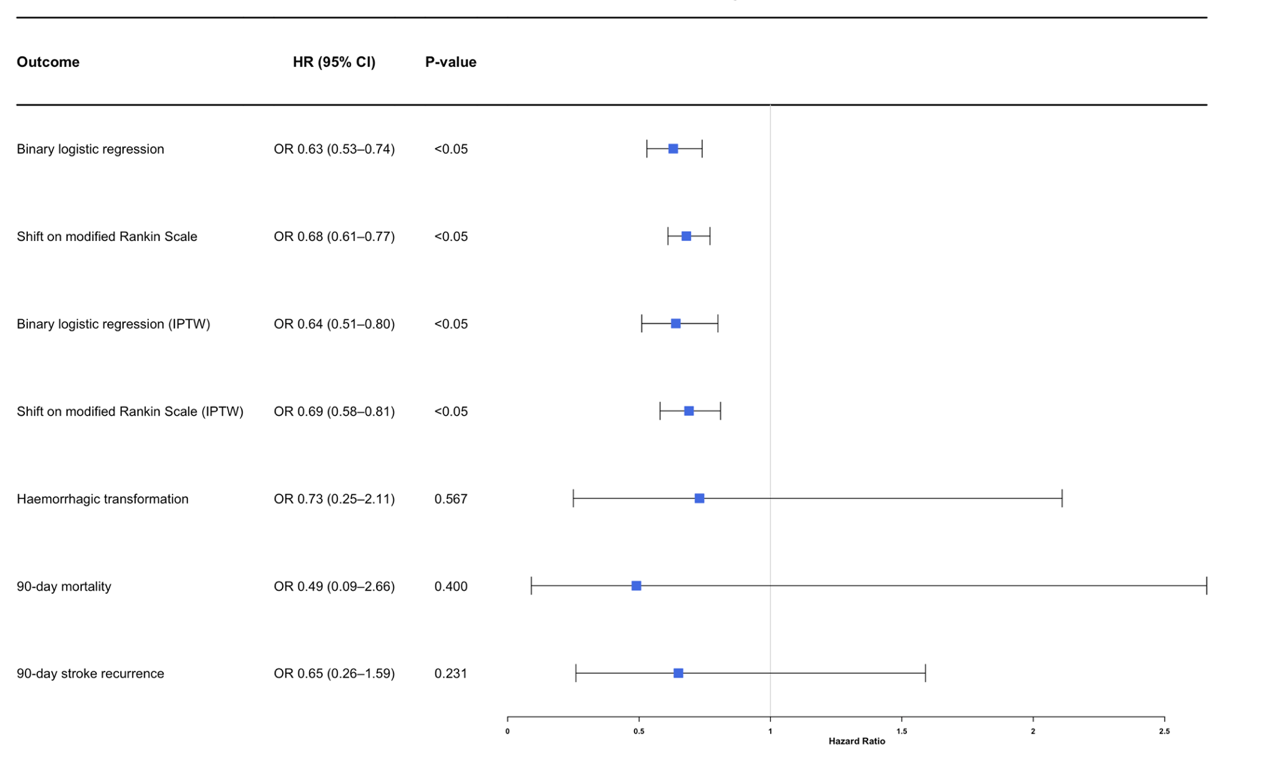


5. Safety outcomes

Among participants in the anisodine hydrobromide group, nine cases of side effects were reported: 3 (0.15%) with dry mouth, 4 (0.19%) with dizziness, 1 (0.05%) with urinary incontinence, and 1 (0.05%) with fatigue (Table 6). No side effects were reported in the best medical treatment group.

At each visit, we compared changes in vital signs (temperature, heart rate, respiratory rate and systolic/diastolic blood pressure) with the last visit within and between group (Table 6-12). Most of the observed variations were not statistically significant, and those with significant difference were considered to have little clinical relevance. The ECG data of most participants (N = 2965, 1549 in anisodine group, 1416 in best medical treatment group) was recorded as well. ECG abnormalities were analyzed using automated systems and validated by trained physicians, including but not limited to sinus tachycardia, atrial fibrillation, paroxysmal supraventricular tachycardia. In the anisodine group, 63.2% of participants had normal ECG, with 11.6% abnormal but without clinical significance, and 25.3% abnormal with clinical significance. In best medical treatment group, the rate of normal, abnormal without clinical significance and abnormal with clinical significance was 67.0%, 10.2%, and 22.9%, respectively (Table 13). However, no significant difference was found between the two groups in terms of ECG test.

Table 6. Side effects caused by anisodine hydrobromide during hospitalization

|  | Anisodine hydrobromide group | | | Best medical treatment group | | | *P* value | | | | |
| --- | --- | --- | --- | --- | --- | --- | --- | --- | --- | --- | --- |
| Dry mouth | 3  4  0  0  1  1  0  0 | | | 0  0  0  0  0  0  0  0 | | | 0.1899 |  |  |  |  |
| Dizziness |  |  |  |  |  |  | 0.1322 |  |  |  |  |
| Facial flushing |  |  |  |  |  |  |  |  |  |  |  |
| Blurred vision / Mydriasis |  |  |  |  |  |  |  |  |  |  |  |
| Urinary incontinence |  |  |  |  |  |  | 0.4439 |  |  |  |  |
| Fatigue |  |  |  |  |  |  | 0.4439 |  |  |  |  |
| Jaundice |  |  |  |  |  |  |  |  |  |  |  |
| Confusion |  |  |  |  |  |  |  |  |  |  |  |
|  |  |  |  |  |  |  |  | |  |  |  |

Table 7. Cardiovascular adverse events during hospitalization

|  | Anisodine hydrobromide group | Best medical treatment group | *P* value |
| --- | --- | --- | --- |
| Heart rate |  |  |  |
| Visit 1 |  |  |  |
| Abnormal | 7(0.36) | 6(0.31) | 0.8091 |
| Visit2 |  |  |  |
| Abnormal | 0(0.00) | 1(0.05) | 0.3123 |
| Visit3 |  |  |  |
| Abnormal | 0(0.00) | 2(0.18) | 0.1415 |
| Visit4 |  |  |  |
| Abnormal | 2(0.10) | 0(0.00) | 0.1613 |
| Systolic blood pressure |  |  |  |
| Visit1 |  |  |  |
| Abnormal | 1276(64.90) | 1279(66.37) | 0.3346 |
| Visit2 |  |  |  |
| Abnormal | 1061(53.97) | 1018(52.86) | 0.4870 |
| Visit3 |  |  |  |
| Abnormal | 553(46.59) | 524(47.68) | 0.6014 |
| Visit4 |  |  |  |
| Abnormal | 825(41.98) | 839(43.54) | 0.3271 |
| Diastolic blood pressure |  |  |  |
| Visit1 |  |  |  |
| Abnormal | 794(40.39) | 824(42.76) | 0.1329 |
| Visit2 |  |  |  |
| Abnormal | 544(27.67) | 533(27.67) | 0.9980 |
| Visit3 |  |  |  |
| Abnormal | 255(21.48) | 247(22.47) | 0.5670 |
| Visit4 |  |  |  |
| Abnormal | 359(18.27) | 403(20.91) | 0.0377 |

Table 8. Analysis of vital signs during hospitalization (body temperature)

|  | Anisodine hydrobromide group | Best medical treatment group | *P* value |
| --- | --- | --- | --- |
| Baseline |  |  |  |
| Mean±SD | 36.47±0.26 | 36.46±0.26 | 0.3137 |
| Visit 2 |  |  |  |
| Mean±SD | 36.47±0.25 | 36.44±0.25 |  |
| Pre–post difference |  |  |  |
| Mean±SD | -0.00±0.32 | 0.02±0.32 |  |
| Within-group comparison of pre–post differences |  |  |  |
| *P* value | 0.6985 | 0.0049 |  |
| Between-group comparison of pre–post differences |  |  | 0.0362 |
| Visit 3 |  |  |  |
| Mean±SD | 36.47±0.27 | 36.45±0.26 |  |
| Pre–post difference |  |  |  |
| Mean±SD | -0.00±0.34 | 0.02±0.35 |  |
| Within-group comparison of pre–post differences |  |  |  |
| *P* value | 0.9504 | 0.0367 |  |
| Between-group comparison of pre–post differences |  |  | 0.1744 |
| Visit 4 |  |  |  |
| Mean±SD | 36.45±0.21 | 36.42±0.23 |  |
| Pre–post difference |  |  |  |
| Mean±SD | 0.02±0.30 | 0.04±0.31 |  |
| Within-group comparison of pre–post differences |  |  |  |
| *P* value | 0.0609 | <0.0001 |  |
| Between-group comparison of pre–post differences |  |  | 0.0002 |

Table 9. Analysis of vital signs during hospitalization (heart rate)

|  | Anisodine hydrobromide group | Best medical treatment group | *P* value |
| --- | --- | --- | --- |
| Baseline |  |  |  |
| Mean±SD | 76.38±10.27 | 76.67±9.91 | 0.2964 |
| Visit 2 |  |  |  |
| Mean±SD | 75.20±8.06 | 75.10±7.79 |  |
| Pre–post difference |  |  |  |
| Mean±SD | 1.20±10.35 | 1.58±10.01 |  |
| Within-group comparison of pre–post differences |  |  |  |
| *P* value | <0.0001 | <0.0001 |  |
| Between-group comparison of pre–post differences |  |  | 0.3010 |
|  |  |  |  |
| Visit 3 |  |  |  |
| Mean±SD | 75.14±7.82 | 75.36±7.61 |  |
| Pre–post difference |  |  |  |
| Mean±SD | 1.51±11.01 | 1.18±10.12 |  |
| Within-group comparison of pre–post differences |  |  |  |
| *P* value | <0.0001 | 0.0029 |  |
| Between-group comparison of pre–post differences |  |  | 0.3478 |
| Visit 4 |  |  |  |
| Mean±SD | 74.90±7.29 | 75.26±7.00 |  |
| Pre–post difference |  |  |  |
| Mean±SD | 1.48±10.53 | 1.39±10.03 |  |
| Within-group comparison of pre–post differences |  |  |  |
| *P* value | <0.0001 | <0.0001 |  |
| Between-group comparison of pre–post differences |  |  | 0.3169 |

Table 10. Analysis of vital signs during hospitalization (respiratory rate)

|  | Anisodine hydrobromide group | Best medical treatment group | *P* value |
| --- | --- | --- | --- |
| Baseline |  |  |  |
| Mean±SD | 18.67±1.46 | 18.68±1.47 | 0.9310 |
| Visit 2 |  |  |  |
| Mean±SD | 18.55±1.49 | 18.60±1.44 |  |
| Pre–post difference |  |  |  |
| Mean±SD | 0.12±1.26 | 0.08±1.31 |  |
| Within-group comparison of pre–post differences |  |  |  |
| *P* value | <0.0001 | 0.0035 |  |
| Between-group comparison of pre–post differences |  |  | 0.7068 |
| Visit 3 |  |  |  |
| Mean±SD | 18.61±1.53 | 18.57±1.44 |  |
| Pre–post difference |  |  |  |
| Mean±SD | 0.14±1.53 | 0.14±1.56 |  |
| Within-group comparison of pre–post differences |  |  |  |
| *P* value | 0.0045 | 0.0089 |  |
| Between-group comparison of pre–post differences |  |  | 0.7512 |
| Visit 4 |  |  |  |
| Mean±SD | 18.53±1.47 | 18.64±1.38 |  |
| Pre–post difference |  |  |  |
| Mean±SD | 0.14±1.32 | 0.04±1.37 |  |
| Within-group comparison of pre–post differences |  |  |  |
| *P* value | <0.0001 | 0.1517 |  |
| Between-group comparison of pre–post differences |  |  | 0.2227 |

Table 11. Analysis of vital signs during hospitalization (systolic blood pressure)

|  | Anisodine hydrobromide group | Best medical treatment group | *P* value |
| --- | --- | --- | --- |
| Baseline |  |  |  |
| Mean±SD | 147.82±21.46 | 149.50±22.02 | 0.0759 |
| Visit 2 |  |  |  |
| Mean±SD | 140.75±16.45 | 140.95±16.15 |  |
| Pre–post difference |  |  |  |
| Mean±SD | 7.07±19.69 | 8.53±19.44 |  |
| Within-group comparison of pre–post differences |  |  |  |
| *P* value | <0.0001 | <0.0001 |  |
| Between-group comparison of pre–post differences |  |  | 0.0221 |
| Visit 3 |  |  |  |
| Mean±SD | 137.91±14.37 | 138.93±14.68 |  |
| Pre–post difference |  |  |  |
| Mean±SD | 9.31±21.15 | 10.18±20.22 |  |
| Within-group comparison of pre–post differences |  |  |  |
| *P* value | <0.0001 | <0.0001 |  |
| Between-group comparison of pre–post differences |  |  | 0.5997 |
| Visit 4 |  |  |  |
| Mean±SD | 135.53±12.15 | 136.43±12.40 |  |
| Pre–post difference |  |  |  |
| Mean±SD | 12.28±20.57 | 13.08±20.21 |  |
| Within-group comparison of pre–post differences |  |  |  |
| *P* value | <0.0001 | <0.0001 |  |
| Between-group comparison of pre–post differences |  |  | 0.5211 |

Table 12. Analysis of vital signs during hospitalization (diastolic blood pressure)

|  | Anisodine hydrobromide group | Best medical treatment group | *P* value |
| --- | --- | --- | --- |
| Baseline |  |  |  |
| Mean±SD | 86.51±13.34 | 87.24±13.84 | 0.1257 |
| Visit 2 |  |  |  |
| Mean±SD | 82.52±10.89 | 82.66±11.21 |  |
| Pre–post difference |  |  |  |
| Mean±SD | 4.00±13.19 | 4.57±12.95 |  |
| Within-group comparison of pre–post differences |  |  |  |
| *P* value | <0.0001 | <0.0001 |  |
| Between-group comparison of pre–post differences |  |  | 0.1175 |
| Visit 3 |  |  |  |
| Mean±SD | 81.09±10.08 | 81.63±10.63 |  |
| Pre–post difference |  |  |  |
| Mean±SD | 4.83±14.18 | 5.02±13.56 |  |
| Within-group comparison of pre–post differences |  |  |  |
| *P* value | <0.0001 | <0.0001 |  |
| Between-group comparison of pre–post differences |  |  | 0.3936 |
| Visit 4 |  |  |  |
| Mean±SD | 80.73±8.44 | 81.14±9.12 |  |
| Pre–post difference |  |  |  |
| Mean±SD | 5.79±13.85 | 6.10±13.03 |  |
| Within-group comparison of pre–post differences |  |  |  |
| *P* value | <0.0001 | <0.0001 |  |
| Between-group comparison of pre–post differences |  |  | 0.3925 |

Table 13. Electrocardiogram during hospitalization

|  | Anisodine hydrobromide group | Best medical treatment group | *P* value |
| --- | --- | --- | --- |
|  |  |  |  |
| Normal | 978(63.14) | 948(66.95) | 0.0926 |
| Abnormal but not clinically significant | 179(11.56) | 144(10.17) |  |
| Abnormal and clinically significant | 392(25.31) | 324(22.88) |  |
| Total | 1549(100.00) | 1416(100.00) |  |

6. ITT and sensitivity analysis

In the supplementary ITT analysis for the primary outcome, similar results were observed. Using multiple imputation, treatment with anisodine hydrobromide was associated with a lower likelihood of good functional outcome (adjusted OR, 0.71; 95% CI, 0.60 to 0.83). Consistent findings were obtained in the sensitivity analysis using the worst-case imputation strategy (adjusted OR, 0.72; 95% CI, 0.63–0.84), supporting the robustness of the results.

Table 14. ITT and worst-case analysis

|  | Adjusted Odds Ratio | 95% CI | *P* value |
| --- | --- | --- | --- |
| Multiple Imputation | 0.71 | 0.60 – 0.83 | <0.0001 |
| Worst-case Imputation | 0.72 | \|  \| \| --- \|   0.63-0.84 | <0.0001 |

7. Subgroup analyses

Exploratory subgroup analyses yielded findings broadly consistent with those of the overall cohort, with anisodine hydrobromide generally associated with a lower risk of unfavorable outcomes across most prespecified subgroups, including age, sex, baseline NIHSS score, hypertension, diabetes, and hyperlipidemia. The treatment effect appeared to remain evident in the large artery atherosclerosis and cardiogenic embolism subgroup. (Figure 3)

Figure 3. Subgroup analysis of primary outcome between Anisodine Hydrobromide and Best Medical Treatment groups.


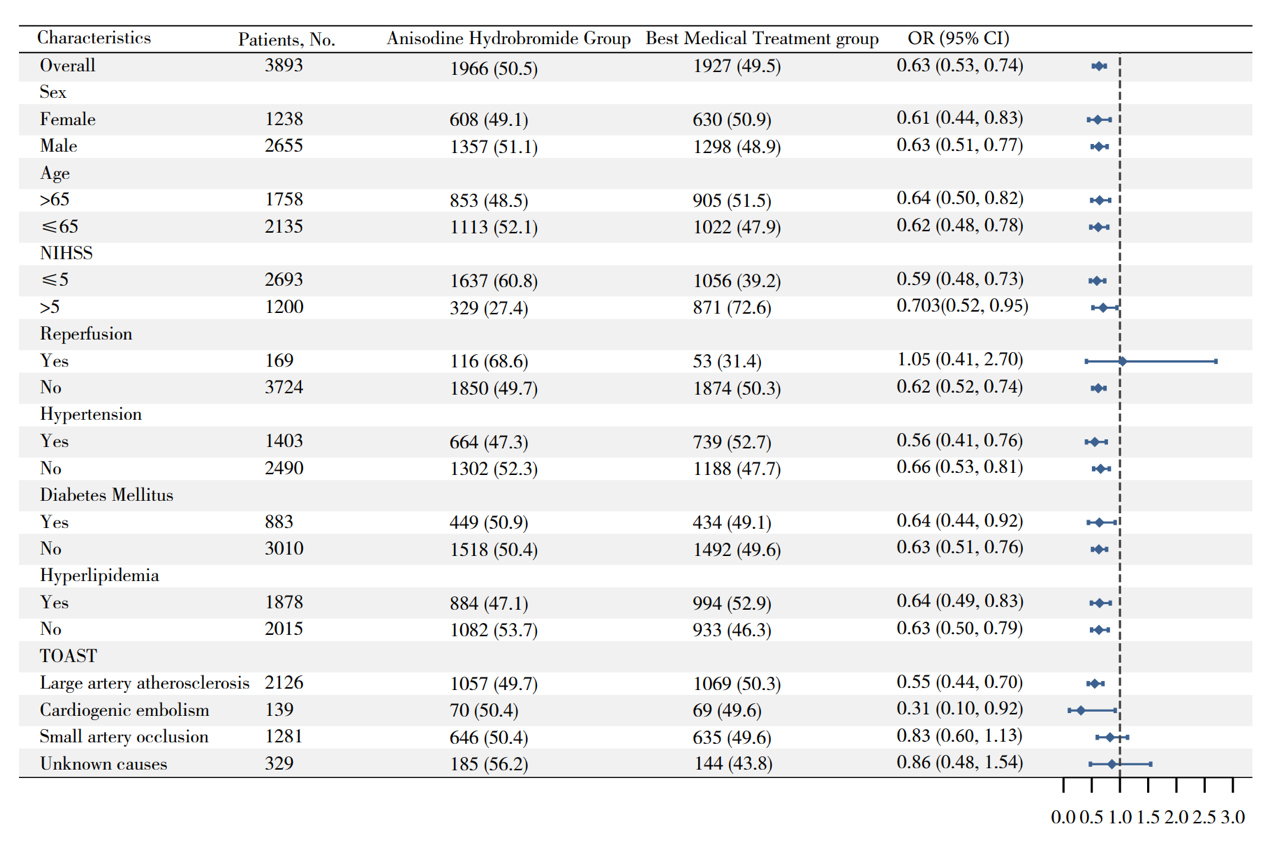


### **Funding**

### **Reference**

1. Neurology C, Society C. Chinese guidelines for diagnosis and treatment of acute ischemic stroke 2018. Chinese Journal of Neurology. 2018 09/08;51:666-82.DOI: <https://dx.doi.org/10.3760/cma.j.issn.1006-7876.2018.09.004>

2. Wu S, Wu B, Liu M, Chen Z, Wang W, Anderson CS, et al. Stroke in China: advances and challenges in epidemiology, prevention, and management. Lancet Neurol. 2019 Apr;18(4):394-405.DOI: <https://dx.doi.org/10.1016/s1474-4422(18)30500-3>

3. Liu L, Li Z, Zhou H, Duan W, Huo X, Xu W, et al. Chinese Stroke Association guidelines for clinical management of ischaemic cerebrovascular diseases: executive summary and 2023 update. Stroke Vasc Neurol. 2023 Dec 29;8(6):e3.DOI: <https://dx.doi.org/10.1136/svn-2023-002998>

4. Ye Q, Zhai F, Chao B, Cao L, Xu Y, Zhang P, et al. Rates of intravenous thrombolysis and endovascular therapy for acute ischaemic stroke in China between 2019 and 2020. The Lancet Regional Health – Western Pacific. 2022;21.DOI: <https://dx.doi.org/10.1016/j.lanwpc.2022.100406>

5. Zhang M, Tian B, Wei W. Effect of compound anisodine on retinal function repair in diabetic retinopathy after panretinal photocoagulation. Zhonghua Shiyan Yanke Zazhi/Chinese Journal of Experimental Ophthalmology. 2015 02/10;33:155-8.DOI: <https://dx.doi.org/10.3760/cma.j.issn.2095-0160.2015.02.012>

6. Wang Y, Wan F, Hu P, He B, Hu Y, Liu Y. Efficacy and safety of anisodine hydrobromide injection for acute ischemic stroke: a systematic review and meta-analysis. Frontiers in Pharmacology. 2023 2023-November-15;14.DOI: <https://dx.doi.org/10.3389/fphar.2023.1290755>

7. Jiang W, Shen J, Du X, Qiu YAN, Zhong J, Ouyang ZHI, et al. Anisodine hydrobromide alleviates oxidative stress caused by hypoxia/reoxygenation in human cerebral microvascular endothelial cells predominantly via inhibition of muscarinic acetylcholine receptor 4. Biocell. 2023 2023/11/08/;47(10):2255-63.DOI: <https://dx.doi.org/https://doi.org/10.32604/biocell.2023.030880>

8. Chen D, Peng C, Xie X, Chen Q, Liu H, Zhang S, et al. Low Dose of Anisodine Hydrobromide Induced Neuroprotective Effects in Chronic Cerebral Hypoperfusion Rats. CNS Neurol Disord Drug Targets. 2017;16(10):1111-9.DOI: <https://dx.doi.org/10.2174/1871527316666171026114043>

9. Zeng Y, Du X, Qiu Y, Jiang W. Anisodine hydrobromide alleviates hypoxia/reoxygenation (H/R)‐induced brain microvascular endothelial cell injury via muscarinic acetylcholine receptor 4. The FASEB Journal. 2021;35.DOI:

10. Palmer R, Dimairo M, Latimer N, Cross E, Brady M, Enderby P, et al. Computerised speech and language therapy or attention control added to usual care for people with long-term post-stroke aphasia: the Big CACTUS three-arm RCT. Health Technol Assess. 2020 Apr;24(19):1-176.DOI: <https://dx.doi.org/10.3310/hta24190>

11. Austin PC, Stuart EA. Moving towards best practice when using inverse probability of treatment weighting (IPTW) using the propensity score to estimate causal treatment effects in observational studies. Stat Med. 2015 Dec 10;34(28):3661-79.DOI: <https://dx.doi.org/10.1002/sim.6607>

12. Chesnaye NC, Stel VS, Tripepi G, Dekker FW, Fu EL, Zoccali C, et al. An introduction to inverse probability of treatment weighting in observational research. Clin Kidney J. 2022 Jan;15(1):14-20.DOI: <https://dx.doi.org/10.1093/ckj/sfab158>

13. Yaghi S, Shu L, Mandel D, Leon Guerrero CR, Henninger N, Muppa J, et al. Antithrombotic Treatment for Stroke Prevention in Cervical Artery Dissection: The STOP-CAD Study. Stroke. 2024;55(4):908-18.DOI: <https://dx.doi.org/doi:10.1161/STROKEAHA.123.045731>
